# Deciphering the Transcription Factor Landscape in Prostate Cancer Progression: A Novel Approach to Understand NE Transdifferentiation

**DOI:** 10.1002/advs.202404938

**Published:** 2025-03-17

**Authors:** Yu Wang, Hui Xue, Xiaohui Zhu, Dong Lin, Zheng Chen, Xin Dong, Junru Chen, Mingchen Shi, Yuchao Ni, Jonathan Cao, Rebecca Wu, Connie Kang, Xinyao Pang, Francesco Crea, Yen‐Yi Lin, Colin C. Collins, Martin E. Gleave, Abhijit Parolia, Arul Chinnaiyan, Christopher J. Ong, Yuzhuo Wang

**Affiliations:** ^1^ Department of Urologic Sciences Faculty of Medicine University of British Columbia Vancouver V5Z 1M9 Canada; ^2^ Vancouver Prostate Centre Vancouver V6H 3Z6 Canada; ^3^ Department of Experimental Therapeutics, BC Cancer Vancouver V5Z 1L3 Canada; ^4^ The First Affiliated Hospital of Jinan University, First Clinical Medical College Jinan University Guangzhou 510632 P. R. China; ^5^ Department of Urology, West China Hospital Sichuan University Chengdu 610041 P. R. China; ^6^ Department of Cell and Systems Biology University of Toronto Toronto M5S 3G5 Canada; ^7^ Cancer Research Group, School of Life Health and Chemical Sciences The Open University Milton Keynes MK7 6AA UK; ^8^ Michigan Center for Translational Pathology Department of Urology University of Michigan Medical School Rogel Cancer Center University of Michigan Hospital Ann Arbor 48109 USA

**Keywords:** adenocarcinoma, De‐differentiation, dormancy, lineage plasticity, NE transdifferentiation, neuroendocrine prostate cancer, transcription factor

## Abstract

Prostate cancer (PCa) stands as a leading cause of cancer‐related mortality among men, with treatment‐induced neuroendocrine prostate cancer (NEPC) posing a challenge as an ARPI‐resistant subtype. The role of transcription factors (TFs) in PCa progression and NEPC transdifferentiation remains inadequately understood, underscoring a critical gap in current research. In this study, an internal Z score‐based approach is developed to identify lineage‐specific TF profiles in prostatic adenocarcinoma and NEPC for a nuanced understanding of TF expression dynamics. Distinct TF profiles for adenocarcinoma and NEPC are unveiled, identifying 126 shared TFs, 46 adenocarcinoma‐TFs, and 56 NEPC‐TFs, validated across multiple cohorts. Gene Ontology is employed to validate their biological and functional roles in PCa progression. Implications are revealed in cell development, differentiation, and lineage determination. Knockdown experiments suggest that lineage‐TFs are functionally important in maintaining lineage‐specific cell proliferation. Additionally, a longitudinal study on NE transdifferentiation highlights dynamic TF expression shifts, proposing a three‐phases hypothesis for PCa progression mechanisms. This study introduces a groundbreaking approach for deciphering the TF landscape in PCa, providing a molecular basis for adenocarcinoma to NEPC progression, and paving the way for innovative treatment strategies with potential impact on patient outcomes.

## Graphical abstract

The study deciphers the landscapes of lineage‐related transcription factors in prostatic adenocarcinoma and neuroendocrine prostate cancer using an innovative approach. These findings, validated across cohorts, highlight TFs' roles in cell development and offer novel therapeutic targets. A longitudinal study on dynamic shift of lineage‐TFs reveals a “three‐phase hypothesis” for NE transdifferentiation, providing crucial insights into prostate cancer progression mechanisms.

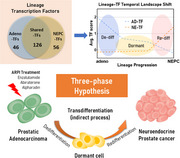

## 1 Introduction

Prostate cancer (PCa) remains a significant public health challenge, being the most commonly diagnosed cancer and the second leading cause of cancer‐related mortality among men in the United States.^[^ ^1^ ^]^ Prostatic carcinogenesis and PCa progression are closely linked to the androgen receptor (AR) signaling pathway, which is targeted by treatments such as androgen deprivation therapy (ADT) and AR pathway inhibitors (ARPIs), including next‐generation ARPIs like Enzalutamide,^[^ ^2^ ^]^ Apalutamide,^[^ ^3^ ^]^ Darolutamide,^[^ ^4^ ^]^ and Abiraterone.^[^ ^5^, ^6^ ^]^ Even though these therapies improve patient outcomes, their use has led to the emergence of a lethal, ARPI‐resistant subtype of prostate cancer known as treatment‐induced neuroendocrine prostate cancer (NEPC), accounting for 10–17% of cases after ARPI therapy.^[^ ^7^, ^8^, ^9^ ^]^

The challenge presented by NEPC, underscored by its resistance to current treatments, brings to light the critical need for a deeper understanding of its development from prostatic adenocarcinoma (PRAD). Insights into the cellular plasticity of PCa, particularly regarding NEPC development, highlight that NEPC may arise from adenocarcinoma through a process known as NE transdifferentiation.^[^ ^10^, ^11^ ^]^ This NE transdifferentiation represents a pivotal lineage transition, enabling tumor cells to evade the constraints of ADT and emerge as an androgen‐independent growth pattern, thereby fostering resistance to therapeutic interventions.^[^ ^12^ ^]^

While adenocarcinoma and NEPC may share certain genetic alterations,^[^ ^13^ ^]^ their transcriptomic landscapes diverge significantly, largely under the influence of their distinctive transcription factors (TFs). Studies across various biological systems have demonstrated that TF expression profiles exhibit lineage‐specific restrictions during cellular differentiation, suggesting that key combinations and interactions among TFs dictate the trajectory of lineage commitment and cellular identity.^[^ ^14^ ^]^ In PCa, this suggests that specialized TF networks are responsible for the gene expression variances observed between adenocarcinoma and NEPC.

Emerging evidence points to the dysregulation of TFs as key oncogenic drivers in PCa progression.^[^ ^15^, ^16^ ^]^ Notably, the AR stands as a central TF in prostatic adenocarcinoma, orchestrating the expression of genes critical for disease pathogenesis.^[^ ^17^ ^]^ Additionally, aberrant expression and activity of TFs from the ETS family, such as ERG and ETV1, has been linked to prostatic adenocarcinoma development.^[^ ^18^, ^19^ ^]^ Moreover, TFs like FOXA1, NKX3.1, and MYC are key regulators of prostatic adenocarcinoma biology,^[^ ^20^, ^21^, ^22^ ^]^ while ASCL1, BRN2, FOXA2, and ONECUT2 play pivotal roles in NEPC lineage identity.^[^ ^23^, ^24^, ^25^, ^26^ ^]^

Recent efforts to characterize TF dynamics in PCa have leveraged high‐throughput genomic and epigenomic approaches to delineate regulatory landscapes across distinct subtypes. Chromatin accessibility and transcriptomic analyses have identified AR‐dependent, Wnt‐driven, and neuroendocrine‐like states, each governed by distinct TF programs.^[^ ^27^ ^]^ By integrating chromatin accessibility with gene expression data, these studies have revealed key TFs that drive lineage plasticity and contribute to treatment resistance. Similarly, analyses of metastatic castration‐resistant prostate cancer (mCRPC) have mapped chromatin accessibility landscapes, highlighting TFs involved in epigenetic reprogramming and therapy resistance.^[^ ^28^ ^]^ Investigations into NEPC heterogeneity further delineate ASCL1‐high and NEUROD1‐high subpopulations, representing two distinct TF‐driven regulatory states.^[^ ^29^ ^]^

While these studies have advanced the understanding of TF‐mediated lineage plasticity, they have primarily focused on specific subtypes or a limited subset of TFs, leaving the broader transcriptional landscape underexplored. This limitation is exacerbated by the inherent sensitivity constraints of high‐throughput technologies, significant background noise, and normalization complexities, which hinder the detection of subtle expression dynamics and low‐abundance TFs. Consequently, despite the 1639 TFs encoded in the human genome,^[^ ^30^ ^]^ only a small fraction has been linked to PRAD and NEPC, leaving many critical regulators unidentified.^[^ ^31^ ^]^ This gap, compounded by the predominant focus on a handful well‐characterized TFs, underscores the need for a more comprehensive approach to fully delineate the TF landscape in PCa, capturing both established and novel regulators implicated in tumor evolution and therapy resistance.

For comparative analysis of transcription factor expression, traditional methods such as fold change‐based comparisons and cross‐sample Z‐score normalization have been widely used.^[^ ^32^ ^]^ However, these approaches present inherent limitations. Fold change analysis does not account for absolute expression levels, often overrepresenting genes with large fold changes but low expression. Conversely, cross‐sample Z‐score ranking normalizes genes relative to the dataset, disregarding their biological relevance within individual samples. This method can disproportionately inflate Z‐scores for highly variable but lowly expressed genes and is further compromised by its sensitivity to outliers.

To address these critical gaps, our study introduces a methodological approach designed for the comprehensive identification and analysis of lineage‐specific TFs in PCa. We applied this innovative approach with the aim of elucidating the temporal dynamics and functional roles of TFs during NE transdifferentiation. We anticipate that our research will not only deepen the understanding of TF‐mediated lineage plasticity and drug resistance in PCa but also unveil novel targets for therapeutic intervention, ultimately contributing to the advancement of personalized cancer treatment strategies and improving patient outcomes.

## 2 Results

### 2.1 An Internal Z Score Approach Reveals TF Profiles in PRAD and NEPC

**Figure 1:**
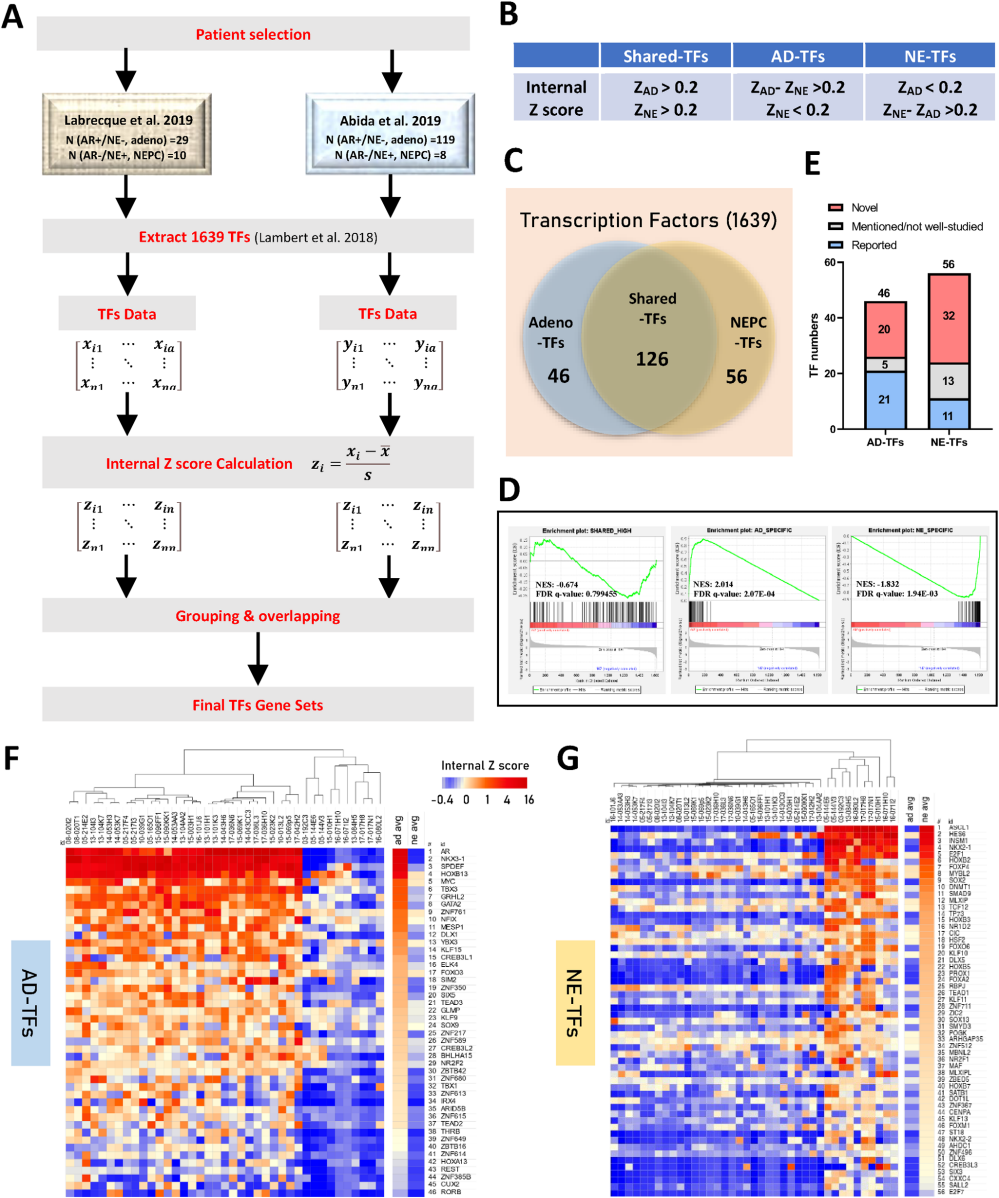
Illustration of the Lineage‐TF Signatures Identification Process. A) Schematic depiction of the internal Z score algorithm developed to discern lineage‐TF signatures. Initial TF expression data matrices were extracted from two distinct clinical cohorts following patient sample selection criteria. Subsequently, internal Z scores were computed to depict the weighted TF expression within each sample. Employing specified criteria, TF gene sets were derived, with final TF lists determined through overlap analysis. B) Criteria for grouping. Z_AD_: the trimmed mean of internal Z score for a TF among PRAD samples. Z_NE_: the trimmed mean of internal Z score for a TF among NEPC samples. C) Summary graph presenting the TF signature identification outcomes. Out of 1639 TFs examined, 126 were identified as shared‐TFs, 46 as AD‐TFs, and 56 as NE‐TFs. D) Gene set enrichment analysis in the Labrecque et al. 2019 cohort indicated enrichment of the shared‐TFs gene set in both PRAD and NEPC patients (NES = −0.674, FDR *q*‐value = 0.79945). The AD‐TFs gene set was enriched solely in PRAD patients (NES = 2.014, FDR *q*‐value = 2.07 × 10^−4^), while the NE‐TFs gene set showed exclusive enrichment in NEPC patients (NES = −1.832, FDR *q*‐value = 1.94 × 10^−3^). E) A comprehensive literature review disclosed that among the 46 AD‐TFs, 21 were previously identified as PRAD‐related regulators, with 20 being novel discoveries. Among the 56 NE‐TFs, 11 were established players in NEPC, 32 were previously unreported, and 13 had been briefly mentioned but not extensively studied. F‐G) Heatmaps exhibiting the signature of F) AD‐TFs and G) NE‐TFs, with hierarchical clustering revealing distinct separation between PRAD and NEPC patients, ranking TFs based on the trimmed mean of internal Z scores within respective lineages. NES: Normalized Enrichment Score. FDR: False Discovery Rate.

As discussed previously, traditional methods such as fold change analysis and cross‐sample Z‐score normalization often misrepresent absolute expression levels and are sensitive to outliers. To address these limitations, we developed an internal Z‐score approach, which calculates the internal Z‐scores within each sample to derive weighted expression profiles before integrating them across datasets. This method minimizes the influence of outliers, preserves biologically meaningful TF expression patterns, and refines lineage‐specific TF identification. As detailed in the Materials and Methods section, our internal Z‐score‐based algorithm (**Figure** **1**A) enables a more accurate characterization of PCa‐related TFs in both PRAD and NEPC.

As PCa is featured as a heterogenous disease, a prior patient sample selection within the cohort was performed to minimize noise, remaining the samples characterized as AR+/NE‐ adenocarcinoma and AR‐/NE+ small cell neuroendocrine carcinoma using two panels of signature genes as reported (Figure S1, Supporting Information).^[^ ^33^ ^]^ Employing the innovative approach mentioned previously, we assessed the internal Z scores of 1639 documented TFs within the same sample, then mapping the individual samples together.^[^ ^30^ ^]^ This method enables identification of distinct TF profiles that delineate either the adenocarcinoma or NEPC lineages. Based on our criteria (Figure 1B), the analysis led to the classification of TFs into three pivotal categories (Figure 1C): (i) 126 shared‐TFs with high internal Z scores in both adenocarcinoma and NEPC (ii) 46 adenocarcinoma‐TFs (AD‐TFs) with high internal Z scores in adenocarcinoma but moderate or low in NEPC (Table S1, Supporting Information), and (iii) 56 NEPC‐TFs (NE‐TFs) (Table S2, Supporting Information).

### 2.2 The Lineage TF Gene Sets are Clinically Relevant and Unveil Novel Candidates for PCa

**Figure 2:**
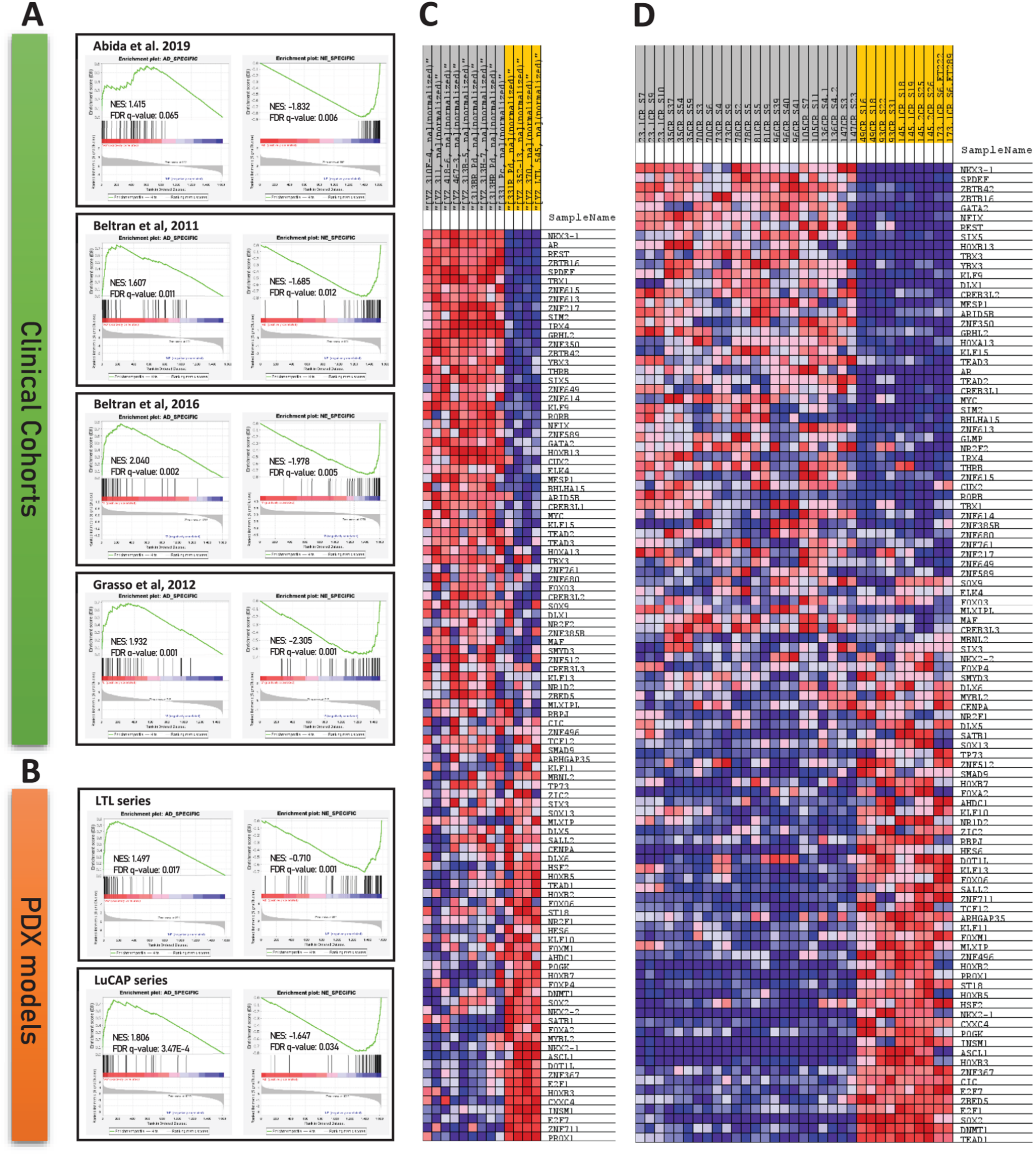
Validation of Lineage‐TF Gene Sets in Clinical Cohorts and PCa PDX Models. (A) GSEA validation of AD‐TFs and NE‐TFs gene sets in Abida et al. 2019 cohort (AD‐TFs NES: 1.415, NE‐TFs NES: −1.832), Beltran et al. 2011 cohort (AD‐TFs NES: 1.607, NE‐TFs NES: −1.685), Beltran et al. 2016 cohort (AD‐TFs NES: 1.607, NE‐TFs NES: −1.685), and Grasso et al. 2012 cohort (AD‐TFs NES: 1.932, NE‐TFs NES: ‐2.305). All cohorts show significant enrichment of lineage‐TFs within respective patient lineages (FDR *q*‐value <0.05). B) GSEA enrichment of AD‐TFs and NE‐TFs in two series of well‐established PCa patient‐derived xenograft (PDX) models: LTL PDXs (AD‐TFs NES: 1.497, NE‐TFs NES: −0.710) and LuCAP PDXs (AD‐TFs NES: 1.806, NE‐TFs NES: −1.647), both with FDR *q*‐value <0.05. Blue–Pink O’ Gram in the Space of the Analyzed GeneSet representing the ratio values of all lineage‐TF referred to 0 in the GeneSet of C) LTL PDX models and D) LuCAP PDX models. NES: Normalized Enrichment Score. FDR: False Discovery Rate.

To demonstrate the broad applicability of our TF gene sets in PCa, we employed gene set enrichment analysis (GSEA) across Labrecque 2019 Cohort.^[^ ^33^ ^]^ The AD‐TFs gene set was enriched exclusively in adenocarcinoma, and the NE‐TFs gene set was only enriched in NEPC samples, while the shared TFs gene set was evenly distributed across both lineages (Figure 1D). In addition, the unbiased hierarchical clustering indicated that the lineage‐specific TFs can distinctly divide the two group of patient samples (Figure 1F,G). The pattern of lineage‐TFs enrichment was consistently replicated in four additional clinical cohorts mentioned previously (**Figure** **2**A), reinforcing the robustness of our findings. Further validation using two well‐known PCa PDX model series, namely the LTL,^[^ ^12^ ^]^ and the LuCAP PDX series,^[^ ^34^ ^]^ demonstrated consistent lineage‐specific TF enrichment (Figure 2B–D).

The comprehensive review of the lineage TFs gene sets revealed a substantial representation of well‐characterized key TFs in both PRAD and NEPC (Figure 1E). Specifically, the AD‐TFs list includes 21 previously reported TFs in adenocarcinoma, with AR occupying the top rank. Among the other 20 AD‐TFs, including NKX3‐1, SPDEF, HOXB13, MYC, GATA2, DLX1, HOXA13, and REST (Figure 1E,F, Table S1, Supporting Information), each has been documented either as functional players or diagnostic biomarkers in prostate cancer.^[^ ^21^, ^35^, ^36^, ^37^, ^38^, ^39^, ^40^, ^41^ ^]^ Similarly, the NE‐TFs list comprises 11 well‐studied TFs NEPC, such as ASCL1, HES6, INSM1, NKX2‐1, E2F1, SOX2, DNMT1, HOXB5, FOXA2, FOXM1, and DOT1L (Figure 1G; Table S2, Supporting Information).^[^ ^23^, ^25^, ^42^, ^43^, ^44^, ^45^, ^46^, ^47^, ^48^, ^49^ ^]^ Additionally, several TFs that have been preliminarily reported but not deepen‐studied in NEPC, such as TEAD1, MAF, NKX2‐2, NR1D2, PROX1, and HOXB7, are also included, suggesting potential key regulators in NEPC. The inclusion of these documented TFs further validates our TF profiles and introduces 20 novel AD‐TFs and 32 novel NE‐TFs (Figure 1E; Tables S1 and S2, Supporting Information), which hold promise as significant players or therapeutic targets for PRAD and NEPC. Notably, certain TFs such as ZNF761, MESP1, CREB3L1, ZNF350, BHLHA15, ZBTB42, ZNF614, and CUX2 exhibit high expression in cancer compared to normal tissue in a pan‐cancer view (Figure S2, Supporting Information), especially in PRAD compared to normal prostate (Figure S3, Supporting Information). These findings suggest potential clinical relevance that warrants further investigation. Furthermore, examination of shared‐high TF profiles reveals the presence of universally recognized oncogenic TFs such as YBX1, FOXA1, JUN, FOS, and STAT3 (Figure S4, Data S1, Supporting Information), suggesting fundamental oncogenic features shared between the two PCa lineages.

### 2.3 AD‐TFs and NE‐TFs Functionally Represent the Corresponding Lineage

**Figure 3:**
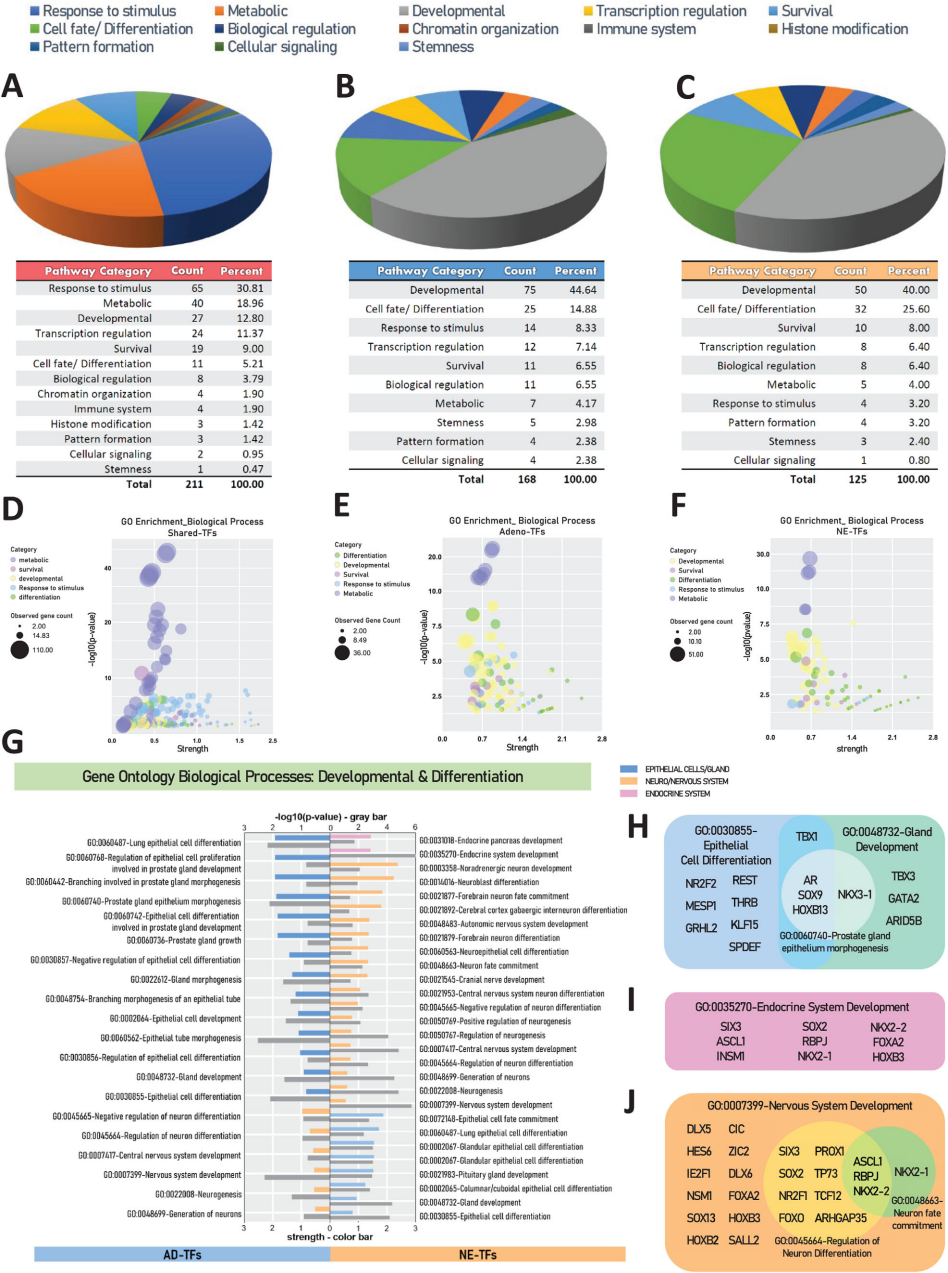
Functional Exploration of Lineage‐TF Gene Sets. Pie charts illustrating the pathway distribution among A) shared‐TFs, B) AD‐TFs, and C) NE‐TFs, with detailed categories and proportions provided in the accompanying charts. Predominant pathway categories in shared‐TFs include those related to stimulus response and metabolism, whereas over half of the enriched pathways in AD‐TFs and NE‐TFs pertain to developmental processes, cell fate and differentiation. Bubble plots D) for shared‐TFs, E) AD‐TFs, and F) NE‐TFs highlight pathway details for the top five categories (metabolic, survival, development, response to stimulus, and cell fate/differentiation), excluding the generic transcription regulation. G) The enriched biological processes (Gene Ontology annotation) associated with developmental and differentiation in AD‐TFs and NE‐TFs exhibit divergent distribution in epithelial gland‐related (Blue bar), neuro‐ and nervous‐related (Orange bar), and endocrine system‐related (Pink bar) pathways. All pathways shown have a *p*‐value <0.01 (Gray‐bar), with the lower x‐axis indicating strength and the upper x‐axis denoting ‐lg(*p*‐value). Detailed TFs involved in key pathways of H) epithelial cell/gland development‐related, I) endocrine system development‐related, and J) nervous system‐related pathways are provided.

To investigate the roles of lineage TFs in cellular processes, bioinformatic analysis was employed to enrich the biological processes in which they are involved. The analysis showed that the shared TFs mostly deal with basic cell activities like reacting to signals, metabolism, and survival. While only a small proportion of pathways were related to developmental processes and cell fate/differentiation (**Figure** **3**A,D, Data S2, Supporting Information), supporting that these shared TFs are universally responsible for the basal metabolism of cells. In contrast, TFs specific to either adenocarcinoma or neuroendocrine cancer were more often involved in pathways that control cell development and differentiation, indicating their potential involvement in lineage‐directing related processes (Figure 3B,C; Data S2, Supporting Information). Specifically, AD‐TFs mainly affect the development of epithelial cell and gland (Figure 3G), with key TFs listed in Figure 3h. NE‐TFs were linked to the development of neural or nervous system and the endocrine system (Figure 3G), with key TFs listed in Figure 3I and Figure 3J. Notably, the AD‐TFs enriched a pathway related to the negative regulation of neuron differentiation, suggesting potential roles of the involved TFs such like SOX9, REST, DLX1, THRB, and FOXO3 play function as suppressors of NE phenotype. In summary, our results show that AD‐TFs and NE‐TFs point to different developmental paths in PCa evolution.

### 2.4 Exploring Novel Lineage‐Specific TFs as Potential Therapeutic Targets in PCa

**Figure 4:**
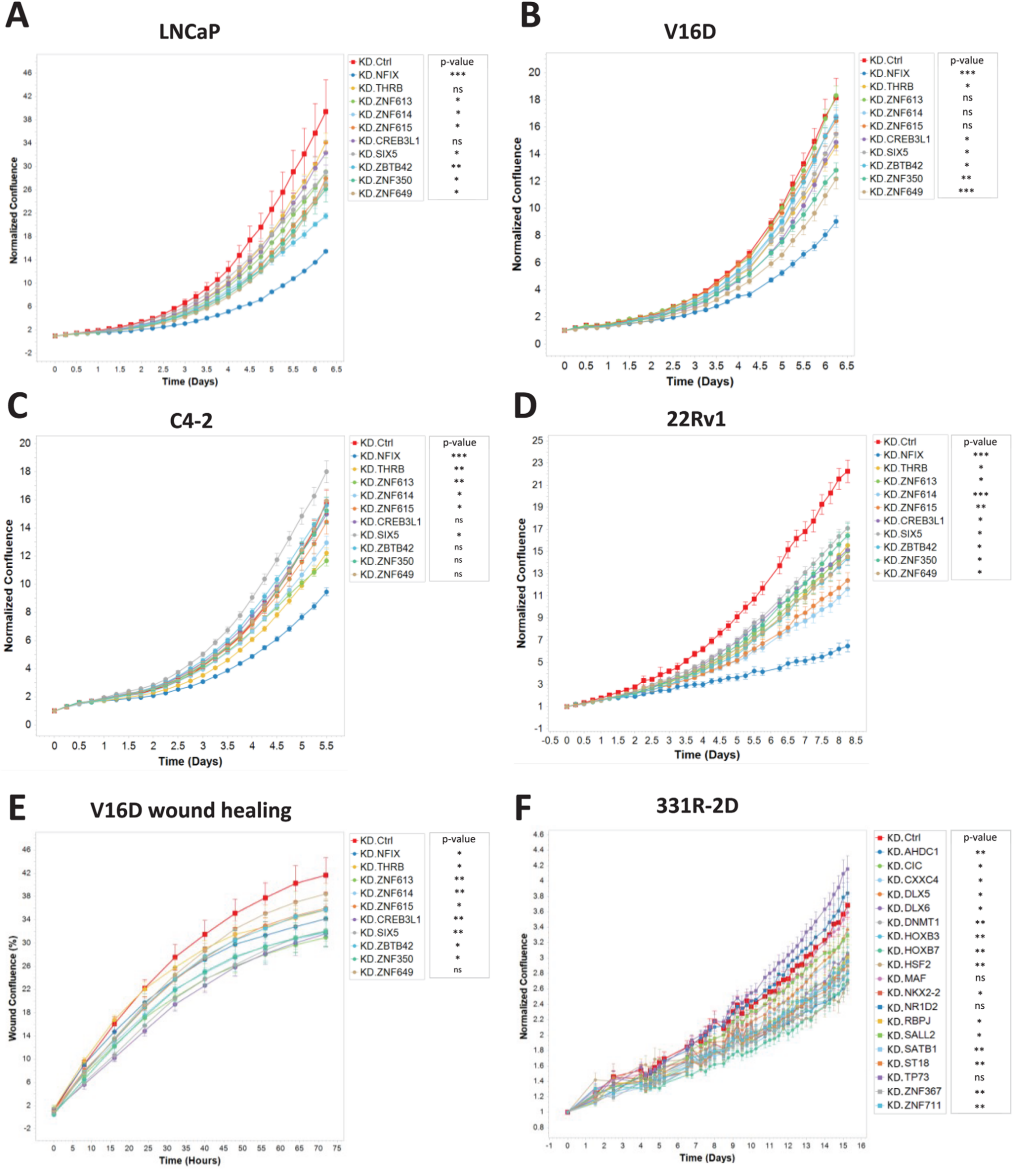
Impact of Lineage‐TFs Depletion on Cell Growth and Migration in Different PCa Cell Lines. The proliferation of human PCa cell lines is compromised when infected with a lineage TF‐targeted sgRNA construct, compared to a control vector. Normalized confluence (relative to the initial point) was employed as a parameter to assess cell proliferation over time, as shown for A) LNCaP cells, B) V16D cells, C) C4‐2 cells, D) 22Rv1 cells, which represent models of PRAD, and E) 331R‐2D cells, which is a cell model derived from NEPC PDX. F) Wound confluence over time was used to evaluate the migration capability of V16D cells following AD‐TF depletion. Data are represented as means ± standard deviation (SD). Statistical analyses comparing to the controls are reflected by *p*‐value: *p < 0.05; **p < 0.01; and ***p < 0.001.

According to the findings of the GO analysis, it is apparent that most lineage‐specific TFs are concurrently implicated in metabolic‐related processes, as reflected by the extremely low *p*‐values and large gene counts (Figure 3E,F). This observation strongly implies that these lineage TFs are strategically positioned to execute vital functions associated with the survival and aggressiveness of cells within their respective lineages. Given the well‐established role of oncogenic transcription factors such as AR and ASCL1 in driving prostate cancer,^[^ ^17^, ^23^ ^]^ our investigation extended to evaluating the contribution of novel lineage‐related TFs to cell growth and aggressiveness. Expanding upon this, we have curated a selection of lineage‐specific TFs that have not been previously documented, thereby enriching the pool of potential therapeutic candidates. We conducted preliminary evaluations of their expression level in TCGA cohorts and Protein Atlas, and select 10 AD‐TF candidates (NFIX, THRB, ZNF613, ZNF614, ZNF615, CREB3L1, SIX5, ZBTB42, ZNF350, ZNF649) and 19 NE‐TF candidates (AHDC1, CIC, CXXC4, DLX5, DLX6, DNMT1, HOXB3, HOXB7, HSF2, MAF, NKX2‐2, NR1D2, RBPJ, SALL2, SATB1, ST18, TP73, ZNF367, ZNF711) for further assessment. Knockdown experiments of AD‐TFs candidates were performed in various PCa adenocarcinoma cell lines, including ADT‐sensitive adenocarcinoma (LNCaP), ADT‐insensitive adenocarcinoma (C4‐2 and V16D), and ADT‐resistant adenocarcinoma (22Rv1) (Knockdown efficacy shown in Figure S5, Supporting Information). We observed that most of the TF knockdowns resulted in inhibition of cell proliferation with varying degrees (**Figure** **4**A–D), and knockdown of AD‐TFs also led to reduced migration in V16D cells (Figure 4F). Additionally, knockdown experiments of NE‐TF candidates were performed in 331R‐2D, a cell line model of NEPC which is developed from NEPC PDX model LTL331R and eligible for in vitro gene manipulation.^[^ ^50^ ^]^ Similarly, knockdown of most NE‐TF candidates inhibited cell proliferation (Figure 4E). These findings highlight the crucial role of these lineage‐specific TFs in tumor survival, suggesting their potential as novel therapeutic targets.

### 2.5 Deciphering the Dynamics of Lineage TFs in NE Transdifferentiation: A Three Phase Hypothesis

**Figure 5:**
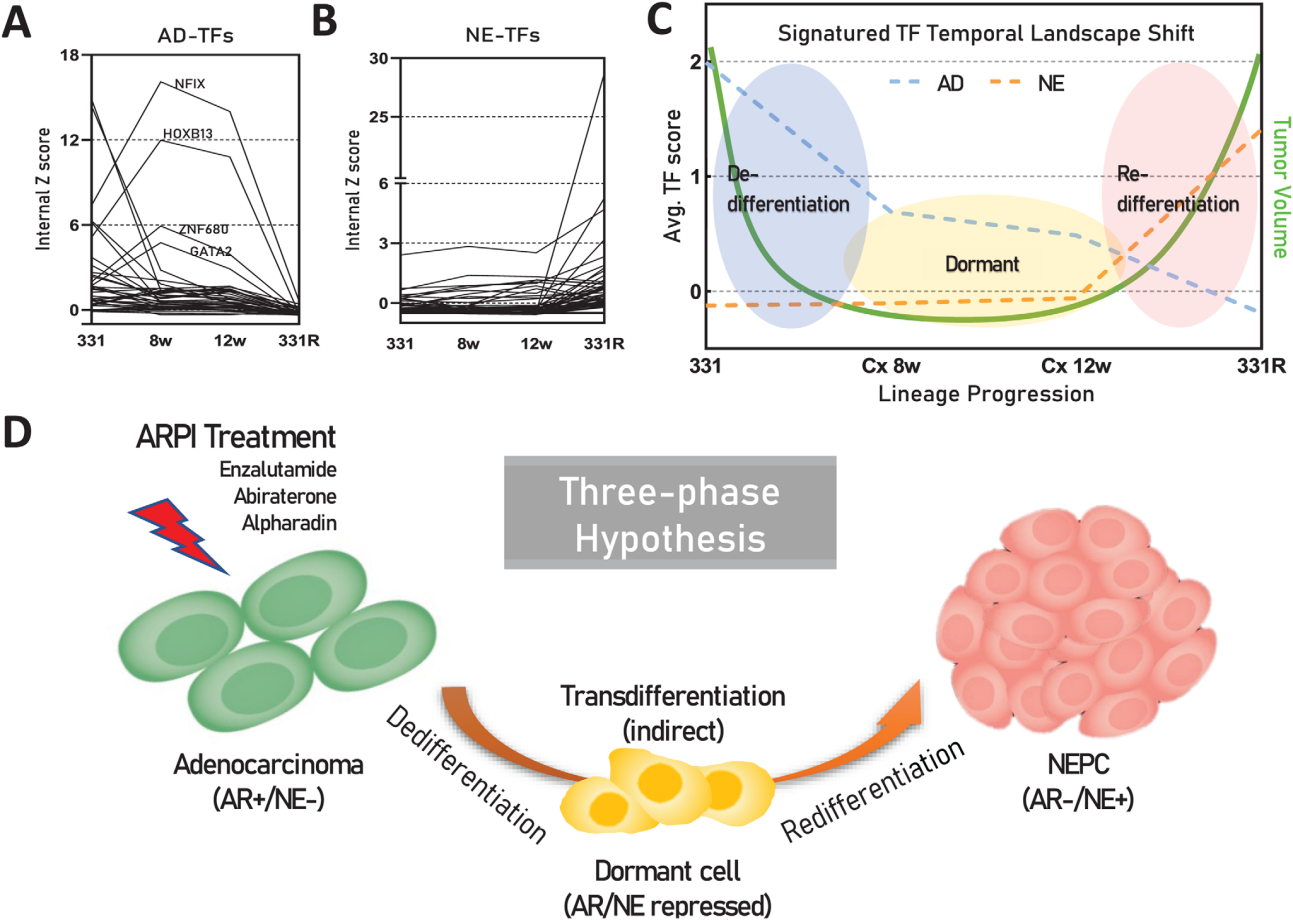
Proposal of the “Three‐Phase Hypothesis”.A) Internal Z scores of AD‐TFs among the 331/331R time series model indicate reduced scores following castration of LTL331, with notable exceptions of NFIX, HOXB13, ZNF680, and GATA2 showing significant increases. *331: LTL331, PRAD; 8w: post‐castrated 8 weeks of LTL331; 12w: post‐castrated 12 weeks of LTL331; 331R: LTL331R, NEPC*. B) Internal Z scores of NE‐TFs among the 331/331R time series model show sustained low scores until the tumor relapse phase. C) Trend of lineage‐TFs shifts along the lineage progression timeline from LTL331 to LTL331R, represented by the average internal Z scores of AD‐TFs (Blue dot line) and NE‐TFs (Orange dot line), delineating the process into three distinct phases: de‐differentiation, dormancy, and re‐differentiation. Green line indicates tumor volume changes. D) Graphic representation of the “Three‐Phase Hypothesis”: AR+/NE‐ prostatic adenocarcinoma cells undergo de‐differentiation under stress from AR pathway inhibition (e.g., enzalutamide, abiraterone, and apalutamide), entering a “dormant” state where both AD and NE features are suppressed. During a prolonged period of dormancy, cells acquire the capability for re‐differentiation, eventually adopting the NE features to become NE+/AR‐ NEPC.

The two groups of lineage TFs, namely AD‐TFs and NE‐TFs, define cellular lineage characteristics. However, how does this TF landscape shift from the adeno lineage to NE lineage is still unknown. Employing the LTL331/331R transdifferentiation model,^[^ ^12^ ^]^ which is designed to recapitulate the development of NEPC from adenocarcinoma, we sought to delineate the shifts in lineage TF expression during this intricate process. By applying the internal Z score algorithm described previously, we observed that the longitudinal changes in TF expression are not stochastic but follow a structured pattern: Most AD‐TFs exhibit a notable reduction within the initial 8 weeks following host castration, persisting at low levels until the relapse phase (**Figure** **5**A). By contrast, NE‐TFs maintain low expression levels until the relapse phase (Figure 5B), indicating the cells may lose their adeno‐feature in a very short period upon ADT while gaining the NE‐feature after a long period when they relapse. This reveals three discernible phases in the transdifferentiation process (Figure 5C), forming the basis for our proposed three‐phase hypothesis.

The first phase, termed the “de‐differentiation phase”, is characterized by the initiation of the loss of AD‐TF features upon androgen deprivation. This stage signifies the adaptive survival and de‐differentiation of PCa cells capable of entering dormancy (i.e., dormancy‐capable cells), whereas most androgen‐dependent PCa cells die of ADT. Subsequently, dormancy‐capable cells transition into a “dormancy phase”, marked by the repression of both AD‐TF and NE‐TF features, representing an intermediate status. After a period of dormancy, during which cancer cells remain inactive, they may eventually acquire the capability to relapse, marking their transition into the “re‐differentiation phase”. This crucial phase is characterized by the reacquisition of specific cell lineage features that had been suppressed, leading toward a cancer relapse (Figure 5D). These features include, but are not limited to, cellular markers associated with proliferation, invasiveness, and potentially, treatment resistance.^[^ ^13^ ^]^ Overall, our longitudinal study of the LTL331/331R model provides a detailed analysis of lineage‐TFs in NE transdifferentiation, offering a comprehensive view of the orchestrated molecular events that occur across three critical phases: de‐differentiation, dormancy, and re‐differentiation. These findings contribute to the elucidation of NEPC development and offer insights into potential therapeutic interventions targeting specific phases of this transdifferentiation process.

### 2.6 TFs in Dormancy: Key Roles in Chromatin Organization and Maintenance of Stemness

**Figure 6:**
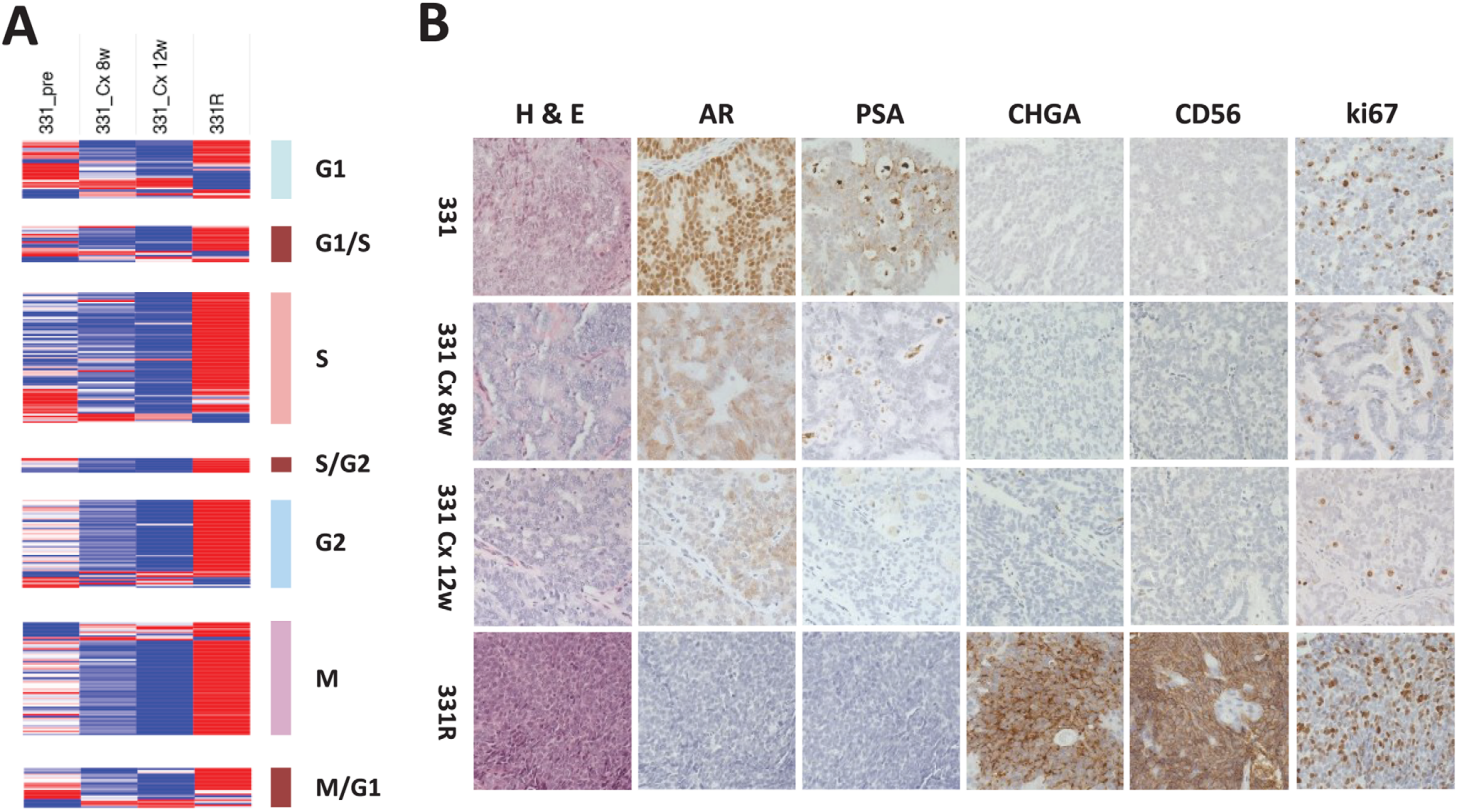
Dormant Features of the LTL331/331R Time Series Model. A) Expression Heatmap of cell cycle‐related genes in LTL331/331R, with genes categorized by related cell cycle phase/checkpoint indicated on the right. G1: G1 phase; S: S phase (synthesis phase); G2: G2 phase; M: M phase (mitotic phase); G1/S: G1/S Checkpoint; S/G2: S/G2 Checkpoint; M/G1: M/G1 checkpoint. B) Immunohistochemical staining of AR pathway signal indicators (AR and prostate‐specific antigen (PSA)), NE phenotype markers (chromogranin A (CHGA) and cluster of differentiation 56 (CD56/NCAM1)), and the proliferation marker Ki‐67, alongside H&E staining depicting tissue original structure. 331: LTL331, PRAD; 331 Cx 8w: post‐castrated 8 weeks of LTL331; 331 Cx 12w: post‐castrated 12 weeks of LTL331; 331R: LTL331R, NEPC.

In the context of PCa, dormancy is a crucial yet poorly understood stage in disease progression, largely due to the absence of appropriate models. Our hypothesis characterized the dormancy phase in NE transdifferentiation by the persistence of a de‐differentiated state with repressed proliferation capability and plays a crucial role in priming for the ensuing re‐differentiation into NEPC. Notably, the established transdifferentiation model LTL331/331R uniquely captures this dormant phase.^[^ ^12^ ^]^ The dormant state of LTL331/331R is characterized by a subdued cell cycle capacity (**Figure** **6**A) and a reduced expression of the proliferation marker Ki67 (Figure 6B), coupled with diminished AR/Prostate‐Specific Antigen (PSA) expression indicative of an adenocarcinoma feature, and suppressed Chromogranin A (CHGA)/Cluster of Differentiation 56 (CD56) expression, which represent NE features (Figure 6B). As previously outlined, most lineage‐TFs adhere to the prevailing trend across the three phases, while a minority display divergent patterns such like NFIX, HOXB13, ZNF680 and GATA2 (Figure 5A). This observation suggests the presence of an additional set of TFs governing the dormancy phase. Consequently, our next objective was to identify TFs associated with dormancy.

**Figure 7:**
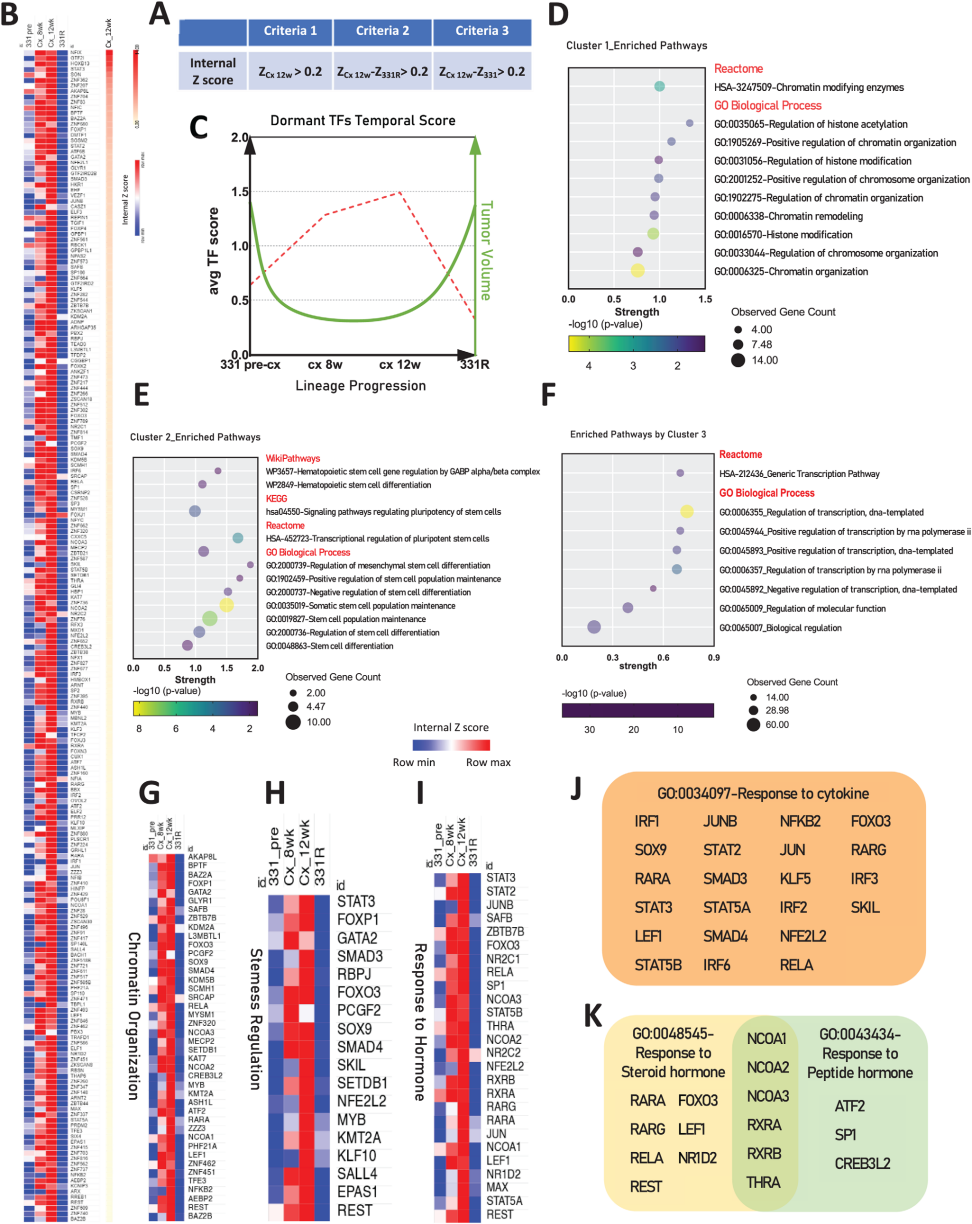
Exploration of dormant‐TFs. A) Criteria for screening of dormant TFs using the internal Z score. Z_Cx 12w_: the TF internal Z score in post‐castrated 12‐week of LTL331. Z_331_/ Z_331R_: the TF internal Z score in LTL331/331R. B) Heatmaps displaying the signature of dormant‐TFs in the LTL331/331R model, with TFs ranked based on Z_Cx 12w._ C) Trend of dormant‐TFs shifts along the lineage progression timeline from LTL331 to LTL331R, depicted by the average internal Z scores (Red dot line), alongside changes in tumor volume (Green line). D) TFs in cluster 1 enriched pathways related to chromatin organization E) TFs in cluster 2 enriched pathways associated with stemness regulation. F) TFs in cluster 3 have enriched pathways related to generic transcription and biological processes. All pathways shown in bubble plots ing *p*‐value < 0.05. All pathways depicted in the bubble plots have a *p*‐value < 0.05. Heatmaps detail expression patterns of key pathways enriched by dormant‐TFs in G) Chromatin Organization, H) Stemness regulation, and I) Response to hormone. Detailed TFs involved in certain pathways shown in (J) Response to cytokine, and K) Response to hormone.

Employing the internal Z score algorithm and screening criteria (**Figure** **7**A), we have identified 213 TFs (Figure 7B; Data S3, Supporting Information) with heightened weighted expression during the dormant phase compared to terminal stages (Figure 7C). The protein‐protein interaction (PPI) network analysis delineated dormant TFs into three distinct functional clusters (Figure S6, Supporting Information). Subsequent bioinformatic analysis unveiled that TFs within cluster 1 are significantly linked with histone modification and chromatin remodeling pathways (Figure 7D), underscoring their potential significance in orchestrating transcriptional program restructuring during the dormant stage, involved TFs are shown in Figure 7G. The prominence of REST within this cluster, recognized as a repressor of the NE phenotype, lends credence to the notion that dormancy represents a repressed and de‐differentiated state. TFs in cluster 2 are implicated in the regulation of stemness, governing pluripotency and stem cell population maintenance (Figure 7E), indicates their potential role in preserving pluripotency for lineage plasticity. A few top‐ranking transcription factors (TFs) within this group (Figure 7H) have been previously associated with the initiation or preservation of dormant states across a variety of biological contexts. For instance, STAT3 has been recognized as a gene associated with dormancy in breast cancer;^[^ ^51^ ^]^ FOXP1 has been shown to enhance characteristics akin to cancer stem cells in ovarian cancer cells;^[^ ^52^ ^]^ FOXO3 is known to regulate cell cycle arrest and metabolism;^[^ ^53^ ^]^ SMADs contributes to tumor dormancy through TGF‐β signaling;^[^ ^54^ ^]^ SOX9 is responsible for maintaining pluripotency,^[^ ^55^ ^]^ and facilitating the reprogramming of prostate cancer cells.^[^ ^56^ ^]^ In addition, the pathway enrichment of cytokine responses (Figure 7J) implies potential intercellular communication via a ligand‐receptor mechanism. For example, members of the Interferon Regulatory Factor (IRF) family, which regulate the Type I interferon system and adaptive immunity, are identified in our analysis.^[^ ^57^ ^]^ Specifically, IRF1, IRF2, IRF3, and IRF6 are included, suggesting their potential roles in protecting the tumor from immune surveillance. This observation underscores the importance of further investigating these factors in the context of tumor immunology. Moreover, a substantial proportion of cluster 2 TFs are associated with pathways responsive to hormones (Figure 7I). The upregulation of retinoic acid (RA) receptors (RAR/RXR genes: RARA, RARG, RXRA, RXRB) provides insights into the potential involvement of RA in NEPC development (Figure 7K). Heightened weighted expression of nuclear receptor coactivators (NCOAs: NCOA1, NCOA2, NCOA3), which have been reported correlated with CRPC progression,^[^ ^58^ ^]^ hinting at the potential underlying mechanism by which dormant cells overcome the hurdle brought by androgen receptor signaling abolishment during NE transdifferentiation. Overall, our findings shed light on the intricate TFs landscape of the dormancy phase during NE transdifferentiation, identifying key TFs and pathways that potentially govern this stage.

## 3 Discussion

Our study introduces a groundbreaking methodological approach for identifying key regulators in PCa, particularly focusing on TFs involved in NE transdifferentiation. Traditional analyses often emphasize differentially expressed genes but can overlook crucial TFs due to their subtle expression changes. By adopting an innovative internal Z score approach, we targeted the weighted expression analysis of 1639 documented TFs in PCa, enhancing our ability to pinpoint pivotal regulators. This methodology, notable for its scientific robustness and ease of application, eliminates common barriers to practical application and is poised for broad adoption across various tumor types and diseases.

Compared to TF‐binding assays such as ChIP‐seq, ATAC‐seq, and CUT&RUN, our gene expression‐based ranking approach offers distinct advantages. While these high‐throughput binding assays provide precise TF‐DNA interaction maps and chromatin accessibility insights, they are inherently limited by antibody availability, sequencing depth requirements, and the context dependency of binding events. Furthermore, TF binding does not always correlate with transcriptional activity, making functional interpretation challenging. Our internal Z‐score approach circumvents these limitations by ranking TFs based on their expression within individual samples while avoiding chromatin accessibility biases. Additionally, our method has a lower technical barrier, as it relies on RNA‐seq data, which is widely available and more scalable across different datasets, enhancing its applicability in diverse cancer studies.

Although our approach provides a robust framework for identifying functionally relevant TFs, it is important to recognize its limitations and potential areas for refinement. Like any gene expression‐based ranking approach, our method relies on gene expression data, which may not fully capture TFs operating within dynamic regulatory feedback loops, such as the Glucocorticoid Receptor (GR). Certain TFs play critical regulatory roles through negative feedback mechanisms, where their expression levels fluctuate dynamically in response to cellular signals. As a result, these TFs may not always rank highly in expression‐based analyses despite being functionally significant. To mitigate this limitation, integrating gene expression‐based ranking with complementary approaches such as functional assays, chromatin accessibility profiling (e.g., ATAC‐seq), and TF activity inference methods (e.g., motif enrichment or TF target analysis) could provide a more comprehensive view of TF dynamics in prostate cancer progression.

Using this method, we identified and characterized two critical sets of TFs that define the distinct characteristics of adenocarcinoma and neuroendocrine lineages. The functional relevance of these lineage‐specific TFs was further demonstrated through knockdown experiments, underscoring their essential roles in maintaining lineage‐specific cell proliferation and providing a wealth of novel candidates for further research and development. Additionally, our longitudinal analysis of NEPC progression from adenocarcinoma revealed trends in the expression of these AD‐TFs and NE‐TFs before, after castration and during relapse, supporting the three‐phase hypothesis of NEPC progression. Using the internal Z score approach, we introduced the concept of dormant‐TFs, which are highly expressed during the dormancy phase.^[^ ^59^, ^60^ ^]^ These dormant‐TFs suggest a potential role in facilitating dormant cell survival and lineage transitions, highlighting the dynamic interplay between different TF sets during NEPC progression.

Historically, the mechanisms of NE transdifferentiation have been poorly understood. Our work contributes to this area by illustrating the dynamic shifts in the lineage TFs landscape across three phases: de‐differentiation, dormancy, and re‐differentiation. We propose that the transition from adenocarcinoma to NEPC is an indirect process, challenging and expanding upon existing hypotheses like epithelial to mesenchymal transition (EMT) and epithelial immune cell‐like transition (EIT).

The initial phase of de‐differentiation, triggered by the stress of androgen deprivation therapy (ADT), highlights a small population of dormancy‐capable cancer cells that evade death by entering dormancy. Targeting this process could reduce the dormant‐capable cell population, enhancing the efficacy of current treatments. During dormancy, cells evade drug and immune responses, a stage we've elucidated by identifying dozens of TFs related to chromatin remodeling and stemness capabilities. Strategies targeting these dormant phase regulators could potentially extend dormancy or achieve lifelong tumor suppression, aiming for non‐recurrence.

In the re‐differentiation phase, cells acquire terminal lineage features, often culminating in cancer relapses. Our findings suggest that intervention strategies should not only focus on treating NEPC but also on preventing progression to this final stage. By targeting early‐stage vulnerabilities, particularly during the de‐differentiation and dormancy phases, we can potentially control tumor development at its most treatable stages.

Overall, the innovative approach and findings of this study significantly advance our understanding of NE transdifferentiation in PCa, offering new directions for research and potential therapeutic interventions. By delineating the complex interplay of TFs across distinct phases of cancer progression, we open new avenues for preventing the aggressive evolution of PCa, aiming to shift the paradigm from managing advanced disease to preventing its onset.

## 4 Experimental Section

### Patient‐Derived Xenografts and Clinical Datasets

All LTL Patient‐Derived Xenografts (PDX) lines were engrafted into NSG mice as previously described.^[^ ^12^ ^]^ This study followed the ethical guidelines stated in the Declaration of Helsinki, specimens were obtained from patients with their informed written consent form following a protocol (#H09‐01628) approved by the Institutional Review Board of the University of British Columbia (UBC). All PDX microarray profiles are available at www.livingtumorlab.com and accessible under the accession number GSE41193 in the GEO database. The LTL331‐castrated tissues and the NEPC‐relapsed LTL331R tissues were harvested at different time points after host castration.^[^ ^61^ ^]^ Transcriptomic analysis of the LTL331/LTL331R time‐series was conducted using RNA‐sequencing data. RNA‐seq profiles for the LTL331/331R castration time‐series have been deposited to the European Nucleotide Archive and are available under the accession number ENA: PRJEB9660. The clinical cohorts utilized in this study include RNA‐seq data from the following sources: Labrecque et al.^[^ ^33^ ^]^ Abida et al.^[^ ^62^ ^]^ Beltran et al.^[^ ^63^ ^]^ Beltran et al.^[^ ^64^ ^]^ Grasso et al.^[^ ^65^ ^]^ and the LuCAP PDX series.^[^ ^34^ ^]^

### TF Score Calculation and TF Gene Set Identification

To calculate the weight expression for a TF, the following steps are performed, the algorithm schematic is shown in Figure 1a.

**1**. Obtain RNA‐seq data matrix of the clinical cohort Labrecque et al.:^[^ ^33^ ^]^ This involves accessing the RNA‐seq data for the clinical cohorts mentioned previously.

**2**. Extract all 1639 TFs: once the data matrix is obtained, extract the expression values for the 1639 transcription factors and obtain the TF data matrix. ***i*** represent the row number, ***a*** represent column number.

(1) $$\left[\begin{array}{ccc} x_{i1} & \cdots & x_{\mathrm{ia}}\\ \vdots & \ddots & \vdots \\ x_{n1} & \cdots & x_{\mathrm{na}} \end{array}\right]$$

**3**. Calculate Column Average $\bar{x}$ and Standard Deviation ***s***: for each sample, calculate the average and standard deviation across all transcription factors.

(2) $$\;\bar{x}=\frac{\sum_{i=1}^{n}x_{i}}{n}s=\sqrt{\frac{\Sigma_{i=1}^{n}{\left(x_{i}-\bar{x}\right)}^{2}}{n-1}}$$

**4**. Internal Z‐score calculation: calculate ***z***_***i***_ for each TF in each sample and obtain the Z score data matrix.

(3) $$\;z_{i}=\frac{x_{i}-\bar{x}}{s}\;\left[\begin{array}{ccc} z_{i1} & \cdots & z_{\mathrm{in}}\\ \vdots & \ddots & \vdots \\ z_{n1} & \cdots & z_{\mathrm{nn}} \end{array}\right]$$

**5**. Calculate Z_ad_ and Z_NE_ using Trimmed Mean ^*^: for each TF, calculate the Trimmed Mean Z‐score of all samples within either adenocarcinoma group (Z_ad_) or NEPC group (Z_NE_), excluding samples with top 10% and bottom 10% Z‐scores within each group to reduce the impact of high‐dispersion samples.

(4) $$Z_{\mathrm{ad}}=\frac{1}{\left|S_{ad}\right|}\;\sum i\in S_{ad}Z_{i},\;\;k_{ad}=\left\lfloor 0.1\times n_{ad}\right\rfloor \;\left|S_{ad}\right|=n_{ad}\;-2k_{ad}$$

(5) $$Z_{\mathrm{NE}}=\frac{1}{\left|S_{NE}\right|}\;\sum i\in S_{NE}Z_{i},\;\;k_{NE}=\left\lfloor 0.1\times n_{NE}\right\rfloor \left|S_{NE}\right|=n_{NE}\;-2k_{NE}$$

^*^: Note that the trimmed mean calculation can be simply performed using the TRIMMEAN function in **Excel** if processing datasets in a spreadsheet format:

(6) $$=\mathrm{TRIMMEAN}\left(A1:\mathrm{An},0.2\right)$$

where **A1:An** represents the sample columns within a group (adenocarcinoma or NEPC), and **0.2** indicates that the top and bottom **10%** of values are excluded before calculating the mean.

**6**. Adjustment of the internal Z‐score threshold for grouping criteria: To effectively distinguish low‐to‐moderate expression TFs from moderate‐to‐high expression TFs, a threshold of 0.2 ^**^ for the internal Z‐score was applied to categorize TFs accordingly.

^**^: The threshold of 0.2 was selected based on RNA‐Seq low‐abundance gene filtering principles, ensuring that TFs classified as highly expressed have biologically meaningful expression levels. An internal Z‐score of 0.2 corresponds to approximately 20 normalized reads, aligning with widely used RNA‐Seq filtering guidelines that recommend threshold of 10–20 normalized reads for low‐abundance genes removal. However, the threshold may be modified based on specific research objectives, including different sequencing platforms, dataset‐specific factors, or tumor heterogeneity considerations. The principle remains to distinguish low‐abundance TFs that lack the representativity of lineage features and reliable expression signals. Empirical validation using well‐characterized high‐ and low‐expressed TFs in specific cancer type should be performed to ensure the adjusted threshold remains biologically meaningful.

**7**. TF gene sets classification: The grouping criteria outlined in Figure 1B were applied to categorize TFs into three distinct gene sets based on their internal Z‐scores (Z_ad_ and Z_NE_):

- **Shared‐TFs**: TFs with Z_ad_ > 0.2 and Z_NE_ > 0.2, indicating moderate‐to‐high expression in both adenocarcinoma and NEPC.
- **AD‐TFs** (Adenocarcinoma High‐TFs): TFs with Z_ad_ – Z_NE_ > 0.2 and Z_NE_ < 0.2, indicating preferential expression in adenocarcinoma but not in NEPC.
- **NE‐TFs** (NEPC High‐TFs): TFs with Z_ad_ < 0.2 and Z_NE_ – Z_ad_ > 0.2, indicating preferential expression in NEPC but not in adenocarcinoma

**8**. Repetition of algorithm for another clinical cohort: following the completion of TF gene set list generation for the initial cohort, the algorithm was repeated for an additional clinical cohort described by Abida et al.^[^ ^62^ ^]^

**9**. Overlap of TF Lists from two cohorts: to finalize the TF gene set lists, an overlap analysis was conducted between the TF lists obtained from the two cohorts. This analysis identified TFs common to both cohorts, thereby consolidating and refining the TF gene sets.

### Gene Set Enrichment Analysis (GSEA)

GSEA (http://software.broadinstitute.org/gsea/index.jsp) was used in this study to determine whether a defined set of genes (e.g., AD‐TFs, NE‐TFs and shared‐TFs) shows significant, concordant differences between two biological phenotypes (e.g., prostatic adenocarcinoma versus NEPC). All GSEA analyses in this study used whole transcriptomic data without expression level cut‐off as expression datasets. Reads counts were used for RNA‐seq data from PDX models and clinical cohorts mentioned previously. A gene set was considered significantly enriched if its normalized enrichment score (NES) has an FDR q below 0.25.

### Gene Ontology (GO) Enrichment Analysis

GO enrichment analysis was performed to elucidate the biological processes associated with the TF gene sets identified in this study. TFs were annotated with GO terms using established databases and functional annotation tools (http://geneontology.org/). Enriched terms were visualized and interpreted to elucidate biological relevance. To facilitate interpretation, enriched GO terms were clustered into functional groups or categories using k‐means clustering.

### Construction of Protein‐Protein Interaction (PPI) Network

The STRING database (https://string‐db.org/) was utilized to construct a PPI network. The analysis was performed by submitting lists of TFs to the STRING database. Interactions among the submitted TFs were retrieved with pre‐defined confidence thresholds to ensure high‐quality interactions with various sources information integrated such as experimental data, computational prediction methods, and curated databases, to generate comprehensive interaction networks. Following the construction of the STRING PPI network, various network analysis techniques were applied to elucidate the functional and structural properties of the network. Measures such as node degree, and clustering coefficient were calculated to assess the topological characteristics of individual nodes within the network.

### Pan‐Cancer Analysis

The online analysis platform TNMplot (https://tnmplot.com/analysis/) was utilized for a comprehensive pan‐cancer analysis of gene expression in normal tissue compared within the paired tumor.^[^ ^66^ ^]^ The database of this tool comprises 56 938 samples, consisting of 33 520 samples from 3180 gene chip‐based studies, 11 010 samples from The Cancer Genome Atlas (TCGA) (including 394 metastatic, 9886 tumorous, and 730 normal samples), 1193 samples from Therapeutically Applicable Research to Generate Effective Treatments (TARGET) (comprising 1 metastatic, 1180 tumorous, and 12 normal samples), and 11 215 normal samples from the Genotype‐Tissue Expression (GTEx) project. Statistical significance was assessed using Mann–Whitney or Kruskal–Wallis tests. False Discovery Rate (FDR) was calculated using the Benjamini–Hochberg method.

### Overall Survival Analysis

cBioPortal (https://www.cbioportal.org/) was utilized to access all available PCa clinical cohorts. cBioPortal is an open‐access resource that provides visualization, analysis, and download of large‐scale cancer genomics datasets. We queried the cohorts for specific genetic alterations and visualized these using the OncoPrint feature. The Kaplan‐Meier method was used to estimate survival functions, and the log‐rank test was used to compare survival distributions between groups (e.g., altered versus unaltered). All statistical analyses were performed using tools of cBioPortal.

### Cell Lines and Cell Culture

The prostatic carcinoma cell lines LNCaP, C4‐2, 22Rv1, and the human embryonic kidney cell line 293T cells were obtained from the American Type Culture Collection (ATCC; Rockville, USA). The castrate resistant prostatic carcinoma cell line V16D was derived through serial xenograft passage of LNCaP cells which was a kind gift from Prof. Amina Zoubeidi laboratory (Vancouver Prostate Centre, Vancouver, Canada).^[^ ^67^ ^]^ The NEPC cell line 331R‐2D were derived through serial xenograft passage of the LTL331R PDX model as previously described.^[^ ^50^ ^]^ Cells were authenticated with the fingerprinting method at Fred Hutchinson Cancer Research Centre (Seattle, USA). Mycoplasma testing was routinely performed at the Vancouver Prostate Centre (Vancouver, Canada). LNCaP, C4‐2, V16D and 22Rv1 cells were maintained in RPMI‐1640 medium (Gibco) containing 10% FBS (Gibco). 293T cells were kept in DMEM medium (Gibco) with 5% FBS. 331R‐2D cells were cultured in RPMI‐1640 medium with supplements as follows: 5% FBS, 10 nmol L^−1^ β‐estradiol (Sigma‐Aldrich), 10 nmol L^−1^ Hydrocortisone (Sigma‐Aldrich), 10 µmol L^−1^ Y‐27632 (Dihydrochloride, Stemcell), 1% Insulin‐Transferrin‐Selenium (Thermo Fisher) and 1% Matrigel (Corning).

### Vector Construction, Virus Production, and Transduction

lentiCRISPR‐v2 was a gift from Feng Zhang (Addgene plasmid #52 961); pMD2.G was a gift from Didier Trono (Addgene plasmid #12 259); psPAX2 was a gift from Didier Trono (Addgene plasmid #12 260). Single‐guide RNAs (sgRNAs) individually targeting 39 TFs (sequences provided in Data S4, Supporting Information) were cloned into lentiCRISPR‐v2 vector as previously described.^[^ ^68^ ^]^ Individual constructed lentiCRISPR‐v2 vectors were co‐transfected with psPAX2 and pMD2.G plasmids into 293T cells using Lipofectamine 3000 (Thermo Fisher) according to the manufacturer's protocol. Culture media containing lentiviruses were collected at 48 and 72 h respectively after virus packaging in 293T cells. Upon filtering, the virus‐contained media were added to the culture media of either LNCaP, C4‐2, V16D, 22Rv1, or 331R‐2D cells, supplemented with 8 µg mL^−1^ polybrene (Sigma‐Aldrich) to enhance transduction efficiency. Puromycin (Gibco) was used at 1 µg mL^−1^ to select for infected cells and maintain stable cell populations.

### Quantitative Reverse Transcription PCR

Total RNA was extracted from cultured cells and patient‐derived xenograft (PDX) tissues using the RNeasy Mini Kit (Qiagen), adhering to the manufacturer's instructions. cDNA synthesis was performed from 1 µg of total RNA utilizing the QuantiTect Reverse Transcription Kit (Qiagen). Quantitative reverse transcription PCR (qRT‐PCR) was conducted on the ABI ViiA 7 Real‐Time PCR system (Applied Biosystems) with TB Green Premix Ex Taq II (Takara Bio). Primer sequences used for the qRT‐PCR assays are provided in Data S5 (Supporting Information). Relative gene expression levels were determined using the 2^(‐ΔΔCt) method, with GAPDH serving as the internal reference gene.

### Label‐Free Cell Proliferation Assay

The Label‐Free Cell Proliferation Assay was conducted using the IncuCyte Live‐Cell Analysis System. Cells were seeded into 96‐well plates in complete medium at specified densities (**Table** **1**), with three replicates per plate. The plates were then subjected to overnight incubation to facilitate cell attachment. Place the 96‐wells plate inside the IncuCyte and allow the plate to warm to 37 °C for 30 min prior to scanning. Set the parameters for Scan type: Standard or Adherent Cell‐by‐Cell (for cell counting); Image channels: Phase; Objective: 10X; Scan interval: every 6 hours. Quantification of cell proliferation across multiple cell types is enabled using IncuCyte AI Confluence Analysis provided within the IncuCyte software package.

**Table 1:** Cell seeding density for proliferation assay in 96‐well plates.

Cell line	LNCaP	C4‐2	V16D	22Rv1	331R‐2D
Seeding density (per well)	2500	2000	2000	4000	20000

### Migration Analysis

The migration assay was conducted using the IncuCyte live‐cell imaging system with Scratch Wound Analysis Software Module. V16D cells were seeded into the 96‐well ImageLock plate (Sartorius) at the density of 5000 per well with three replicates per plate, then allowed the cells grow to reach confluence. Wounds in all wells were simultaneously created using the 96‐pin IncuCyte WoundMaker according to the manufacturer's protocol. After washing by PBS, complete medium was added into the plate which was then placed inside the IncuCyte. Set the parameters for Scan type: Scratch Wound; Image channels: Phase; Objective: 10X; Scan interval: every 8 hours. The data was then analyzed by the integrated metric of wound confluence using IncuCyte software package.

### Immunohistochemistry (IHC) Analysis and Antibodies

The harvested xenografts of LTL331/331R time series model were bisected along their longest dimension, fixed in 10% neutral‐buffered formalin, and subsequently embedded in paraffin. Tissue sections were prepared from the formalin‐fixed, paraffin‐embedded samples, and routine hematoxylin and eosin (H&E) staining, as well as IHC, were performed following established protocols as previously described,^[^ ^12^ ^]^ the primary antibodies including a rabbit monoclonal anti‐AR antibody (1:100, Abcam), a rabbit monoclonal anti‐PSA antibody (1:200, Abcam), a monoclonal mouse anti‐human Ki‐67 antibody (1:25, Dako), a rabbit monoclonal anti‐CHGA antibody (1:500, Abcam) and a rabbit monoclonal anti‐CD56 antibody (1:2000, Abcam).

### Statistical Analysis and Data Representation

Statistical analysis was done using the Graphpad Prism software and the IncuCyte software package. The student t‐test was used to analyze statistical significance between groups in discrete measurements, whereas Two‐way ANOVA was used for continuous measurements. Heatmaps were constructed using Morpheus tool (https://software.broadinstitute.org/morpheus/). The distance measure utilized in hierarchical clustering analysis was the Euclidean distance which served as the basis for clustering similar samples together in a hierarchical manner, facilitating the identification of underlying patterns and relationships within the dataset. Any difference with P values lower than 0.05 is regarded as statistically significant, with *p < 0.05; **p < 0.01; ***p < 0.001; and ****p < 0.0001. Graphs show pooled data with error bars representing SEM obtained from at least three replicates and SD of values from clinical samples.

## Conflict of Interest

The authors declare no conflict of interest.

## Author Contributions

Conceptualization: Y.W. and Y.Z.W. Methodology: Y.W. and Y.Z.W. Xenograft studies: D.L., H.X., X.D. H&E and IHC: R.W. Advice on experiments: C.K. and F.C. Data acquisition: C.C. and Y.L. Bioinformatic analysis: Y.W., Y.N., X.Z., Z.C., X.P. In vitro experiment: Y.W., J.C., M.S., J.C. Data interpreted: Y.W., X.Z., Y.Z.W., A.P., A.C. Supervision: Y.Z.W., M.E.G., A.C., and C.J.O. Writing—original draft: YW, YZW, A.P., and A.C. Writing—review & editing: Y.W., Y.Z.W., M.E.G., AC, and C.J.O.

## Supporting information



Supporting Information

Supporting Information

Supporting Information

Supporting Information

Supporting Information

Supporting Information

## Data Availability

The data that support the findings of this study are available in the supplementary material of this article.
