# Supplementary material for: Deciphering the Transcription Factor Landscape in Prostate Cancer Progression: A Novel Approach to Understand NE Transdifferentiation
Source: Adv Sci (Weinh). 2025 Mar 17;12(20):2404938. doi: 10.1002/advs.202404938 (PMC12120771; doi:10.1002/advs.202404938)
Supplement: Supplementary file 1 — Supporting Information [file ADVS-12-2404938-s003.docx]

Supporting Information

Deciphering the Transcription Factor Landscape in Prostate Cancer Progression: A Novel Approach to Understand NE Transdifferentiation

Yu Wang, Hui Xue, Dong Lin, Xiaohui Zhu, Zheng Chen, Xin Dong, Junru Chen, Mingchen Shi, Yuchao Ni, Jonathan Cao, Rebecca Wu, Connie Kang, Xinyao Pang, Francesco Crea, Yen-Yi Lin, Colin C. Collins, Martin E. Gleave, Abhijit Parolia, Arul Chinnaiyan, Christopher J. Ong, Yuzhuo Wang*

**This file includes:**

Tables S1 and S2

Figures S1 to S6

**Other Supplementary Materials for this manuscript include the following:**

**Supplemental Material as Excel files:**

**Supplemental Data S1.** List of shared-TFs

**Supplemental Data S2.** GO process enrichments of lineage TFs and shared TFs

**Supplemental Data S3.** List of dormant-TFs

**Supplemental Data S4.** List of guide sequence for CRISPR ko

**Supplemental Data S5_**List of primers for qRT-PCR

Table S1. AD- TFs list

| Rank | Gene Name | Z_AD_ | Z_NE_ | Z_AD_-Z_NE_ | p-value | Novelty in PRAD |
| --- | --- | --- | --- | --- | --- | --- |
| **1** | AR | 12.2120 | -0.3449 | 12.5570 | 1.95E-08 | reported |
| **2** | NKX3-1 | 11.3254 | -0.3374 | 11.6628 | 1.08E-13 | reported |
| **3** | SPDEF | 10.3354 | -0.1559 | 10.4913 | 1.72E-15 | reported |
| **4** | HOXB13 | 5.7601 | 0.1648 | 5.5953 | 5.12E-11 | reported |
| **5** | MYC | 1.5457 | 0.1358 | 1.4099 | 6.30E-05 | reported |
| **6** | TBX3 | 1.5455 | -0.1175 | 1.6630 | 2.95E-10 | Not-well studied |
| **7** | GRHL2 | 1.4861 | -0.0843 | 1.5704 | 7.38E-11 | reported |
| **8** | GATA2 | 1.3587 | -0.1617 | 1.5203 | 2.90E-06 | reported |
| **9** | ZNF761 | 0.9096 | 0.1428 | 0.7668 | 6.63E-04 | Novel |
| **10** | NFIX | 0.9035 | 0.0221 | 0.8814 | 1.73E-05 | Not-well studied |
| **11** | MESP1 | 0.8833 | -0.1193 | 1.0026 | 9.54E-07 | Novel |
| **12** | DLX1 | 0.7125 | -0.1923 | 0.9048 | 1.91E-07 | reported |
| **13** | YBX3 | 0.6914 | 0.0706 | 0.6209 | 2.78E-05 | Novel |
| **14** | KLF15 | 0.6472 | -0.1362 | 0.7833 | 1.13E-06 | Novel |
| **15** | CREB3L1 | 0.5059 | -0.1678 | 0.6737 | 1.25E-04 | Not-well studied |
| **16** | ELK4 | 0.4767 | -0.0564 | 0.5331 | 2.25E-04 | reported |
| **17** | FOXO3 | 0.4733 | 0.1399 | 0.3334 | 6.91E-03 | reported |
| **18** | SIM2 | 0.4712 | -0.2193 | 0.6905 | 3.82E-02 | reported |
| **19** | ZNF350 | 0.4459 | -0.2428 | 0.6887 | 9.00E-07 | Novel |
| **20** | SIX5 | 0.4451 | -0.1577 | 0.6028 | 9.88E-07 | Novel |
| **21** | TEAD3 | 0.4406 | -0.0285 | 0.4690 | 5.11E-04 | Novel |
| **22** | GLMP | 0.3787 | -0.0229 | 0.4016 | 1.30E-05 | Novel |
| **23** | KLF9 | 0.3513 | -0.0372 | 0.3885 | 1.34E-06 | reported |
| **24** | SOX9 | 0.3342 | 0.0031 | 0.3312 | 3.87E-03 | reported |
| **25** | ZNF217 | 0.3327 | -0.2333 | 0.5660 | 3.43E-11 | Not-well studied |
| **26** | ZNF589 | 0.2979 | -0.0467 | 0.3446 | 4.08E-03 | Novel |
| **27** | CREB3L2 | 0.2933 | -0.0669 | 0.3603 | 4.18E-03 | reported |
| **28** | BHLHA15 | 0.2814 | -0.1272 | 0.4086 | 1.78E-02 | Novel |
| **29** | NR2F2 | 0.2180 | -0.0508 | 0.2689 | 6.79E-05 | reported |
| **30** | ZBTB42 | 0.2168 | -0.2798 | 0.4966 | 5.08E-11 | Novel |
| **31** | ZNF680 | 0.2133 | -0.2197 | 0.4330 | 5.44E-04 | Novel |
| **32** | TBX1 | 0.1840 | -0.2885 | 0.4725 | 5.35E-03 | reported |
| **33** | ZNF613 | 0.1736 | -0.2936 | 0.4672 | 1.08E-06 | Novel |
| **34** | IRX4 | 0.1684 | -0.3740 | 0.5424 | 6.12E-05 | reported |
| **35** | ARID5B | 0.1583 | -0.1922 | 0.3505 | 4.21E-06 | reported |
| **36** | ZNF615 | 0.1546 | -0.2037 | 0.3584 | 3.36E-05 | Novel |
| **37** | TEAD2 | 0.1516 | -0.1969 | 0.3485 | 6.83E-04 | Novel |
| **38** | THRB | 0.0307 | -0.3466 | 0.3773 | 1.93E-07 | Not-well studied |
| **39** | ZNF649 | 0.0108 | -0.2812 | 0.2920 | 7.00E-07 | Novel |
| **40** | ZBTB16 | -0.0013 | -0.2894 | 0.2881 | 2.08E-08 | reported |
| **41** | ZNF614 | -0.0351 | -0.2430 | 0.2080 | 1.39E-05 | Novel |
| **42** | HOXA13 | -0.0556 | -0.2919 | 0.2363 | 3.07E-03 | reported |
| **43** | REST | -0.0727 | -0.3126 | 0.2399 | 3.14E-08 | reported |
| **44** | ZNF385B | -0.0812 | -0.2986 | 0.2173 | 6.56E-03 | Novel |
| **45** | CUX2 | -0.0836 | -0.2843 | 0.2007 | 4.46E-03 | Novel |
| **46** | RORB | -0.1338 | -0.3667 | 0.2329 | 2.31E-03 | Novel |

Z_AD_: Trimmed mean of internal Z score among prostatic adenocarcinoma patient samples.

Z_NE_: Trimmed mean of internal Z score among NEPC patient samples.

**Table S2. NE-TFs list**

| Rank | Gene Name | Z_AD_ | Z_NE_ | Z_AD_-Z_NE_ | p-value | Novelty in NEPC |
| --- | --- | --- | --- | --- | --- | --- |
| **1** | ASCL1 | -0.3113 | 13.3250 | -13.6363 | 4.96E-06 | reported |
| **2** | HES6 | 0.1490 | 9.8258 | -9.6768 | 1.14E-02 | reported |
| **3** | INSM1 | -0.2500 | 8.4701 | -8.7202 | 1.24E-03 | reported |
| **4** | NKX2-1 | -0.3104 | 3.3342 | -3.6446 | 6.94E-04 | reported |
| **5** | E2F1 | 0.0997 | 3.3173 | -3.2176 | 5.43E-04 | reported |
| **6** | HOXB2 | -0.1753 | 1.7893 | -1.9646 | 4.56E-02 | Not-well studied |
| **7** | FOXP4 | 0.1801 | 1.2400 | -1.0600 | 4.15E-03 | Novel |
| **8** | MYBL2 | 0.1600 | 1.0318 | -0.8718 | 3.59E-03 | Novel |
| **9** | SOX2 | -0.3137 | 0.8435 | -1.1572 | 1.96E-03 | reported |
| **10** | DNMT1 | -0.0475 | 0.7760 | -0.8235 | 1.07E-04 | reported |
| **11** | SMAD9 | -0.1989 | 0.6720 | -0.8708 | 1.03E-01 | Novel |
| **12** | MLXIP | 0.1097 | 0.6580 | -0.5483 | 8.07E-03 | Novel |
| **13** | TCF12 | 0.0567 | 0.5982 | -0.5416 | 4.53E-02 | Not-well studied |
| **14** | TP73 | -0.2890 | 0.5477 | -0.8367 | 1.45E-03 | Novel |
| **15** | HOXB3 | -0.2326 | 0.5462 | -0.7787 | 1.11E-02 | Novel |
| **16** | NR1D2 | 0.0932 | 0.5092 | -0.4160 | 1.31E-01 | Not-well studied |
| **17** | CIC | 0.0211 | 0.5051 | -0.4840 | 4.34E-04 | Novel |
| **18** | HSF2 | 0.0474 | 0.4865 | -0.4391 | 2.59E-02 | Novel |
| **19** | FOXO6 | -0.2331 | 0.4735 | -0.7066 | 2.04E-03 | Novel |
| **20** | KLF10 | 0.0285 | 0.4608 | -0.4323 | 2.82E-03 | Novel |
| **21** | DLX5 | -0.2335 | 0.4577 | -0.6912 | 4.98E-03 | Novel |
| **22** | HOXB5 | -0.2862 | 0.4191 | -0.7053 | 1.49E-02 | reported |
| **23** | PROX1 | -0.3157 | 0.4024 | -0.7180 | 3.49E-03 | Not-well studied |
| **24** | FOXA2 | -0.3133 | 0.3990 | -0.7123 | 1.50E-03 | reported |
| **25** | RBPJ | 0.1648 | 0.3954 | -0.2306 | 1.41E-01 | Not-well studied |
| **26** | TEAD1 | -0.1730 | 0.3779 | -0.5510 | 8.94E-03 | Not-well studied |
| **27** | KLF11 | -0.0602 | 0.3417 | -0.4019 | 1.31E-02 | Novel |
| **28** | ZNF711 | -0.3046 | 0.3394 | -0.6440 | 1.89E-02 | Novel |
| **29** | ZIC2 | -0.2266 | 0.3335 | -0.5601 | 1.18E-02 | Novel |
| **30** | SOX13 | -0.0070 | 0.3107 | -0.3176 | 3.57E-02 | Novel |
| **31** | SMYD3 | -0.2104 | 0.2968 | -0.5072 | 1.26E-02 | Not-well studied |
| **32** | POGK | 0.0339 | 0.2940 | -0.2601 | 6.08E-02 | Novel |
| **33** | ARHGAP35 | 0.0622 | 0.2809 | -0.2186 | 2.35E-02 | Novel |
| **34** | ZNF512 | 0.0108 | 0.2490 | -0.2382 | 9.55E-02 | Novel |
| **35** | MBNL2 | -0.0943 | 0.2485 | -0.3428 | 3.13E-02 | Novel |
| **36** | NR2F1 | -0.2132 | 0.2182 | -0.4314 | 1.28E-01 | Not-well studied |
| **37** | MAF | -0.0331 | 0.2116 | -0.2447 | 1.44E-01 | Novel |
| **38** | MLXIPL | -0.1885 | 0.1959 | -0.3844 | 1.36E-01 | Novel |
| **39** | ZBED5 | -0.0274 | 0.1747 | -0.2021 | 3.29E-03 | Novel |
| **40** | HOXB7 | -0.1434 | 0.1715 | -0.3149 | 2.01E-02 | Not-well studied |
| **41** | SATB1 | -0.2469 | 0.1616 | -0.4085 | 3.40E-02 | Not-well studied |
| **42** | DOT1L | -0.0946 | 0.1510 | -0.2457 | 5.39E-06 | reported |
| **43** | ZNF367 | -0.2213 | 0.1488 | -0.3701 | 5.96E-03 | Novel |
| **44** | CENPA | -0.1485 | 0.1298 | -0.2783 | 1.01E-02 | Not-well studied |
| **45** | KLF13 | -0.1843 | 0.1212 | -0.3055 | 4.76E-03 | Novel |
| **46** | FOXM1 | -0.1091 | 0.0992 | -0.2083 | 5.05E-02 | reported |
| **47** | ST18 | -0.3298 | 0.0885 | -0.4183 | 4.48E-03 | Not-well studied |
| **48** | NKX2-2 | -0.2573 | 0.0856 | -0.3429 | 5.79E-02 | Not-well studied |
| **49** | AHDC1 | -0.1802 | 0.0802 | -0.2604 | 1.34E-02 | Novel |
| **50** | ZNF496 | -0.1486 | 0.0665 | -0.2151 | 9.58E-04 | Novel |
| **51** | DLX6 | -0.3171 | 0.0437 | -0.3609 | 2.06E-02 | Novel |
| **52** | CREB3L3 | -0.3136 | 0.0241 | -0.3376 | 2.86E-01 | Novel |
| **53** | SIX3 | -0.3280 | 0.0228 | -0.3508 | 6.94E-04 | Novel |
| **54** | CXXC4 | -0.3124 | -0.0163 | -0.2961 | 3.71E-03 | Novel |
| **55** | SALL2 | -0.2570 | -0.0290 | -0.2280 | 6.65E-03 | Novel |
| **56** | E2F7 | -0.3062 | -0.1041 | -0.2022 | 1.59E-02 | Novel |

Z_AD_: Trimmed mean of internal Z score among prostatic adenocarcinoma patient samples.

Z_NE_: Trimmed mean of internal Z score among NEPC patient samples.

**Fig. S1**


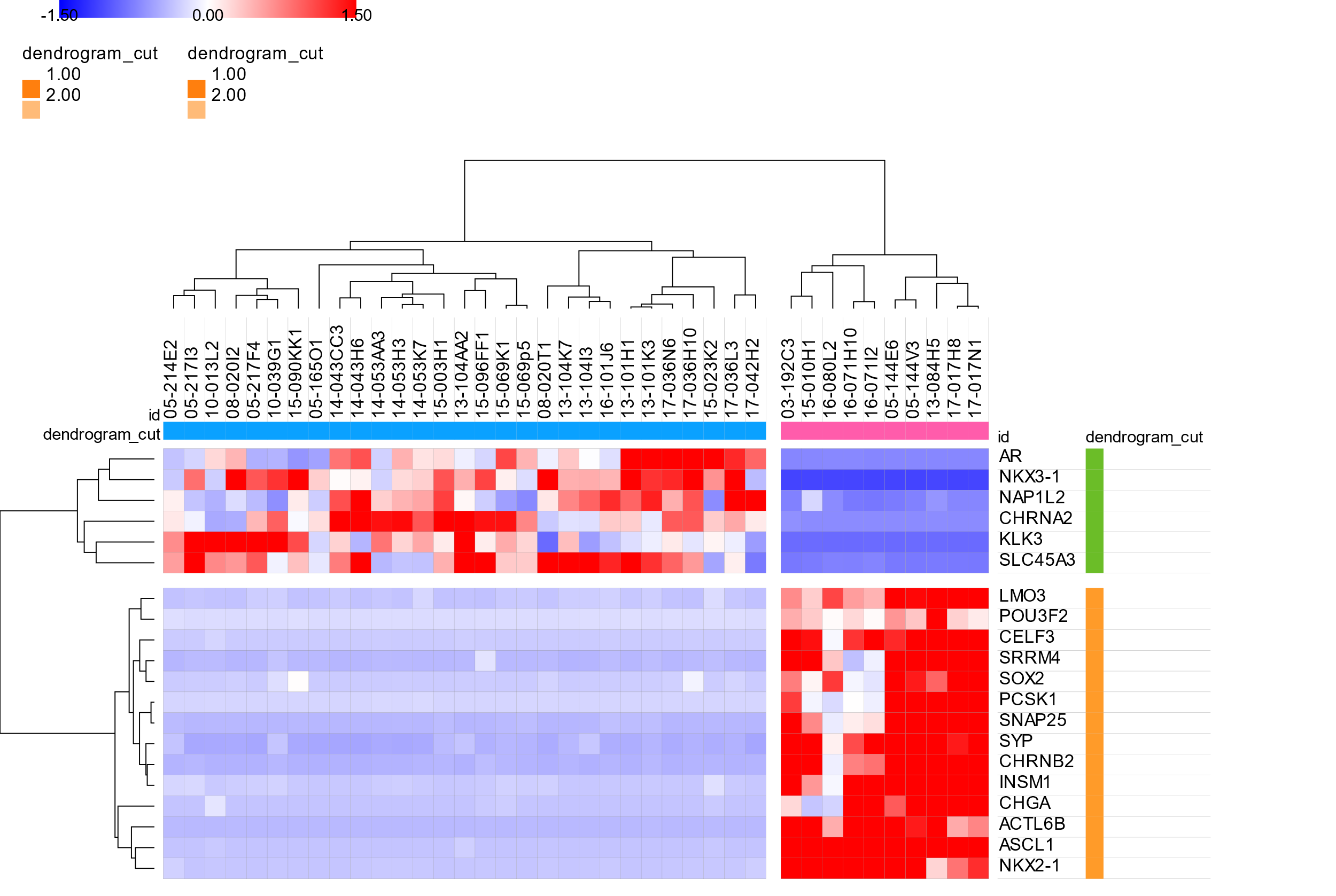

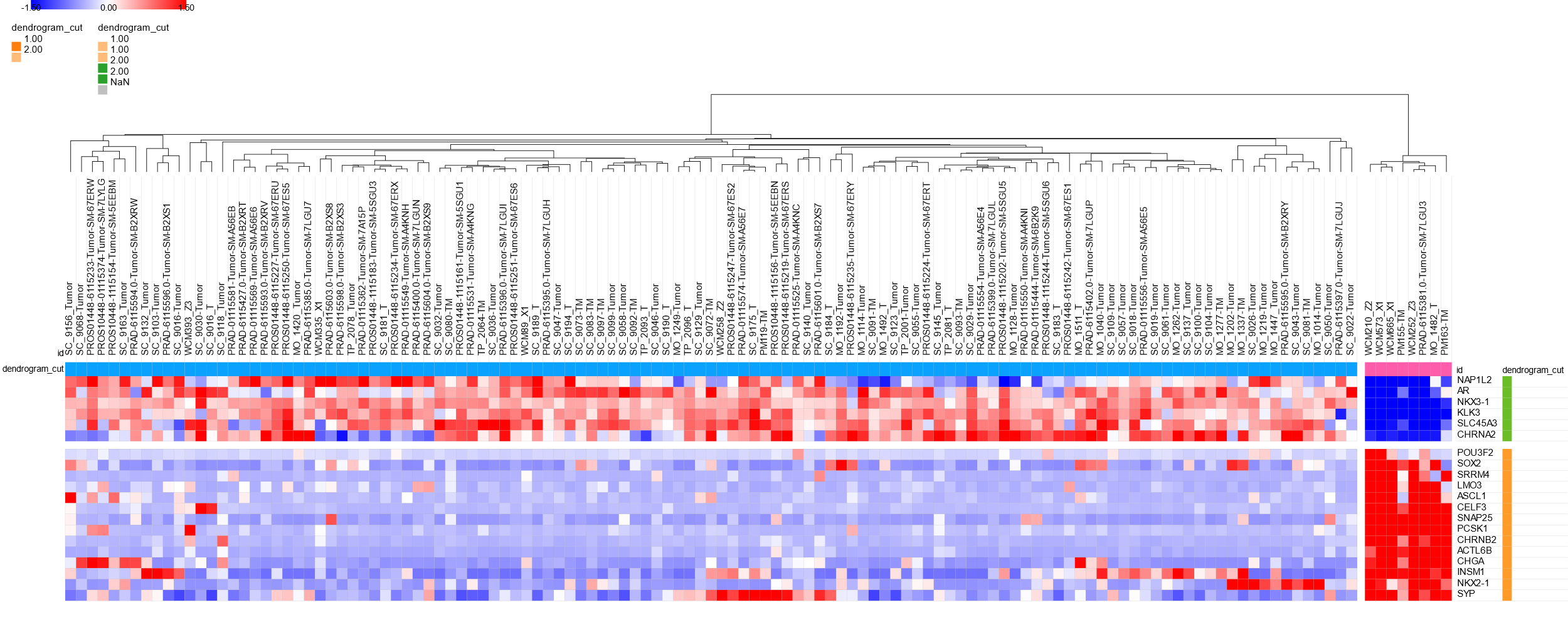


**A**

**B**


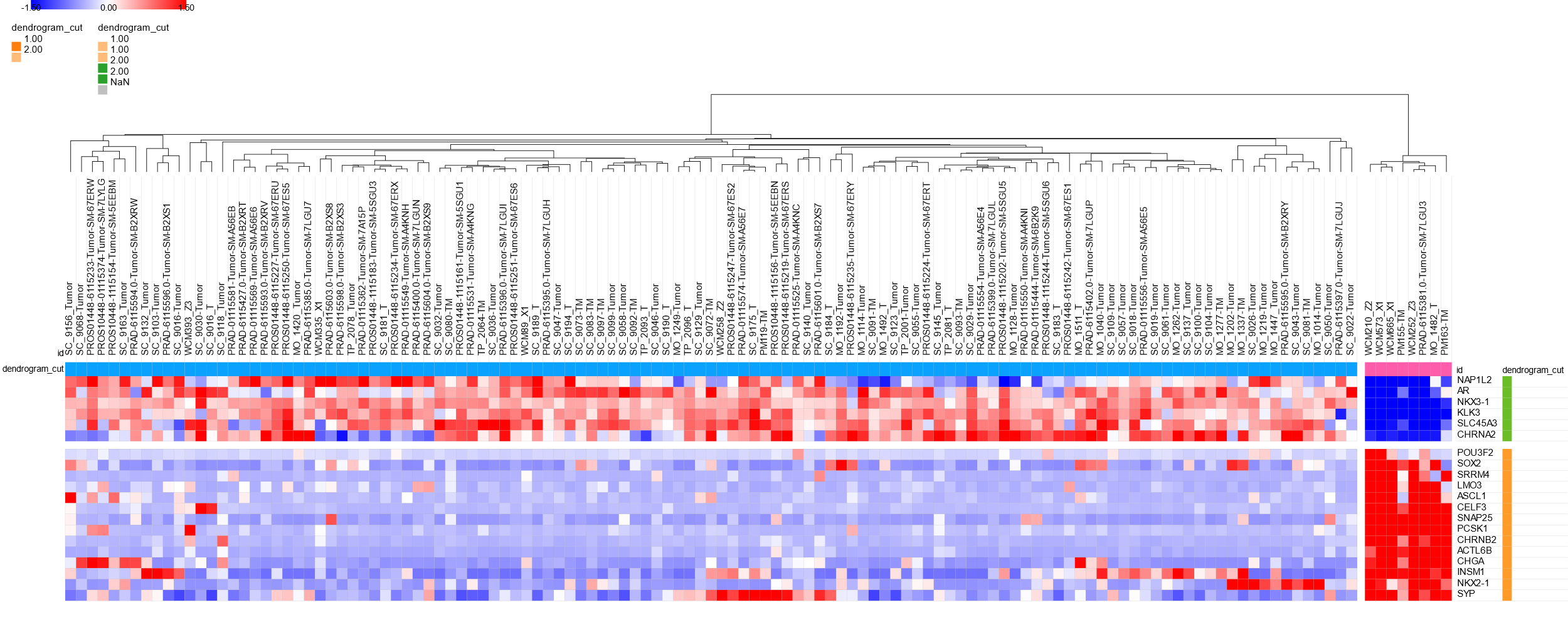


dendrogram cut

Signature Panel of AR

Signature Panel of NE

**Figure S1. Patient selection of the TFs-identification cohorts.** The heatmaps display patient samples for the identification of lineage-TFs in (**A**) the Labrecque et al. 2019 Cohort, and (**B**) the Abida et al. 2019 Cohort, using signature gene panels of AR and NE. Specifically, only patients classified as AR+/NE- for prostate adenocarcinoma (PRAD) and AR-/NE+ for neuroendocrine prostate cancer (NEPC) were included.

**Figure. S2**


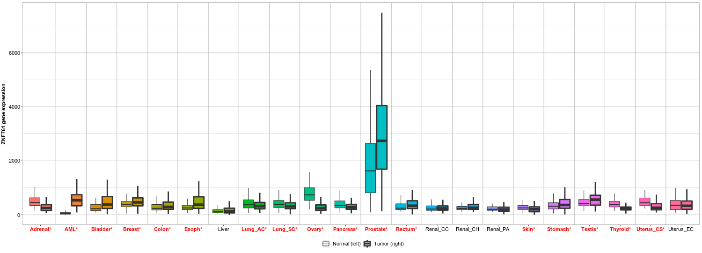


**ZNF761**


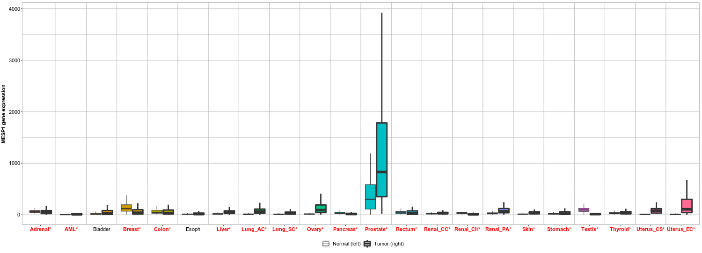


**MESP1**

**CREB3L1**


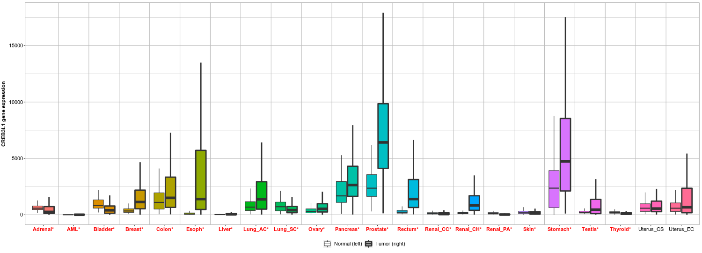


**ZNF350**


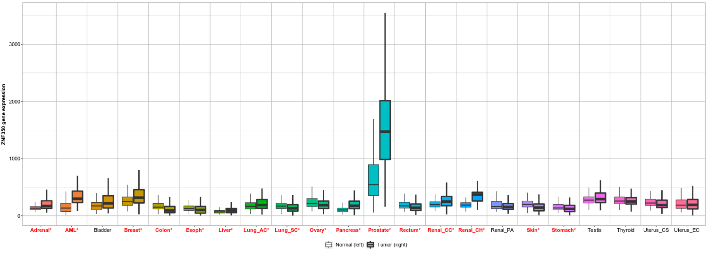


**BHLHA15**


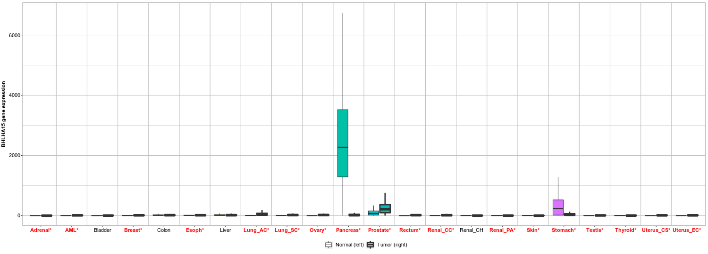


**ZBTB42**


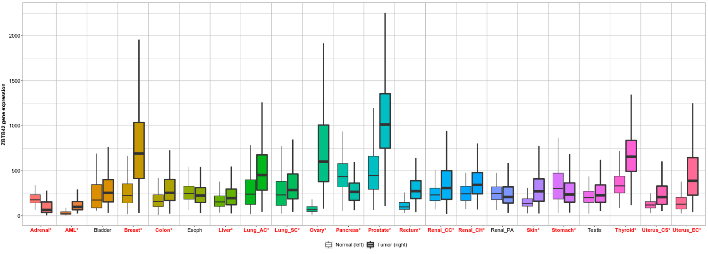


**ZNF614**


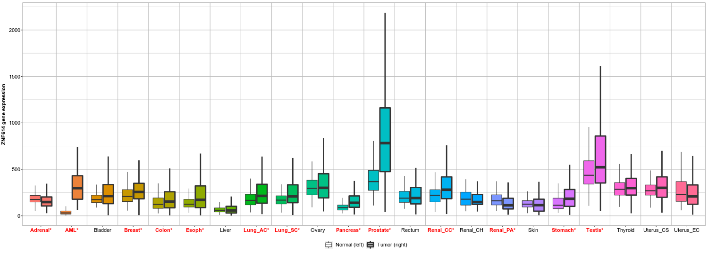


**CUX2**


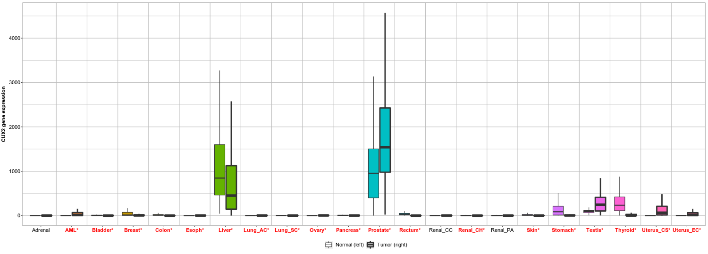


**Pan-cancer analysis： Normal vs. Tumor**

**Figure S2. Pan-cancer analysis of AD-TFs.** Expression Boxplots of AD-TFs including ZNF761, MESP1, CREB3L1, ZNF350, BHLHA15, ZBTB42, ZNF614, CUX2 in 22 common cancer types. The left box in each type of cancer indicates gene expression in corresponding normal tissue, the right box indicates the expression in tumor. Significant differences by a Mann–Whitney U test are marked with red color (* p < 0.01).

**Figure. S3**

**Prostatic adenocarcinoma**


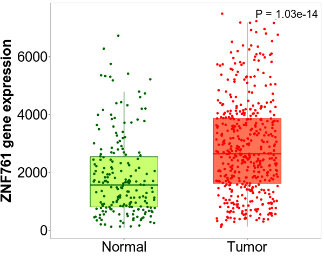

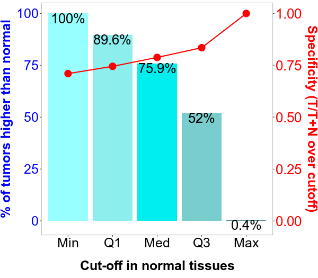


**ZNF761**


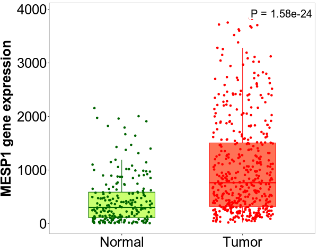

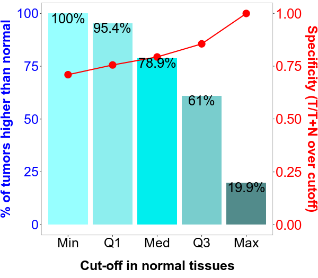


**MESP1**

**CREB3L1**


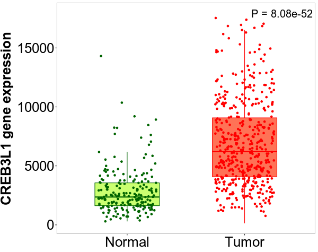

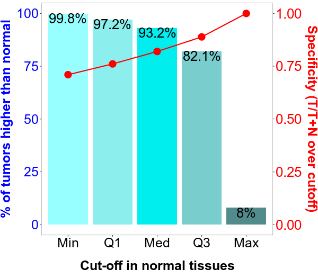


**ZNF350**


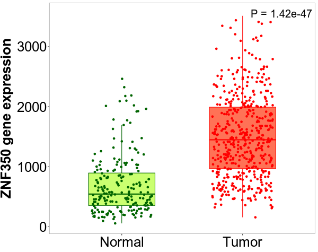

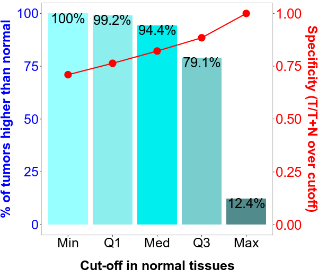


**BHLHA15**


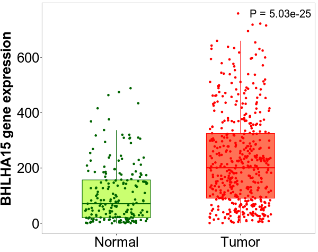

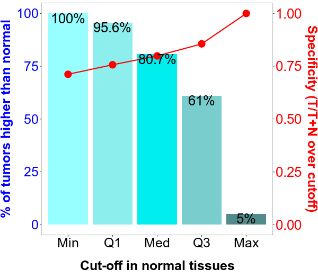


**ZBTB42**


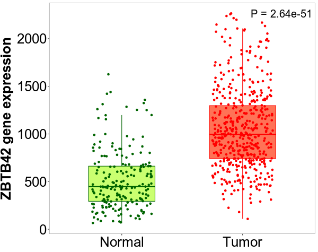

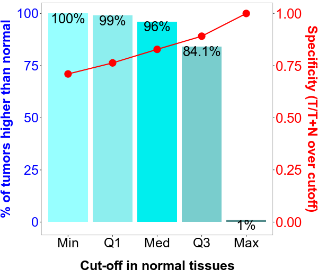

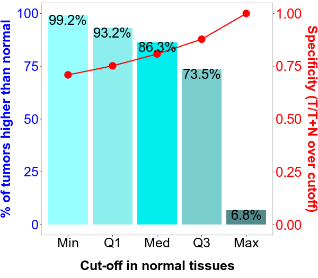


**ZNF614**


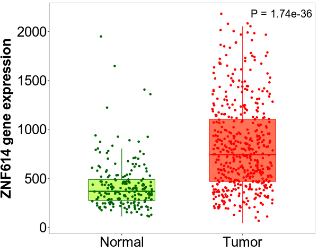


**CUX2**


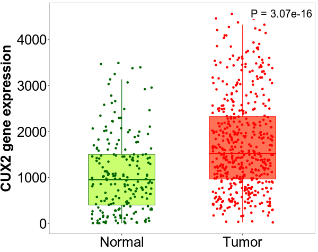

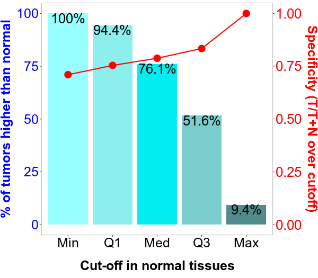


**Figure S3. The clinical relevance of AD-TF candidates in PRAD.** Boxplots indicate the gene expression in paired normal prostate tissue (Green box) or PRAD (Red box). The bars represent the proportions of tumor samples exhibiting higher expression of the selected gene compared to normal samples at each of the quantile cutoff values (minimum, 1st quartile, median, 3rd quartile, maximum). Specificity is calculated by dividing the number of tumor samples with values over each given cutoff by the sum of tumor and normal samples. For fold changes exceeding 1, those 'over' were considered instead of those 'below'.

**Figure. S4**

**Figure S4. Heatmaps exhibiting the signature of shared-TFs.** The heatmap visualizes the internal Z-scores of TFs shared by both PRAD and NEPC, ranking TFs based on the average internal Z-score across the two lineages.


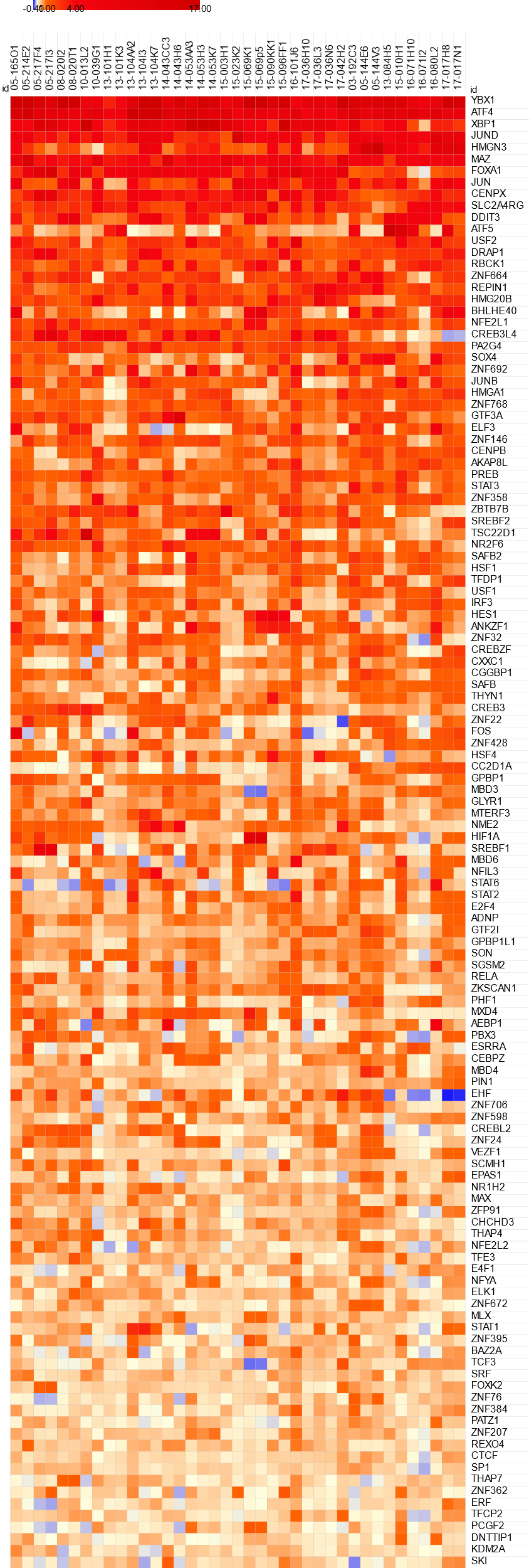

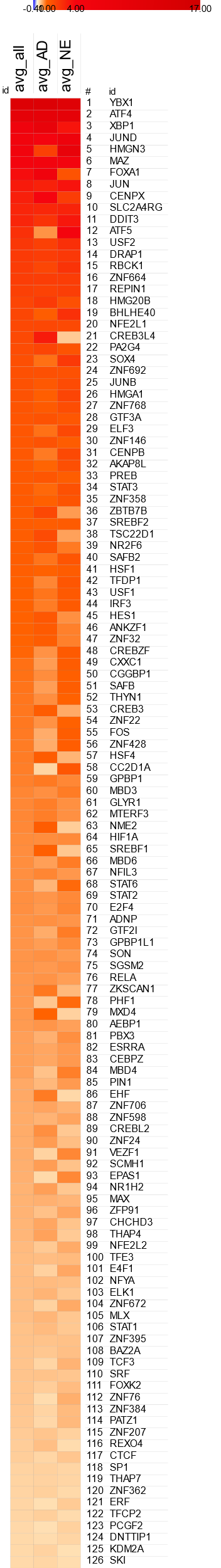

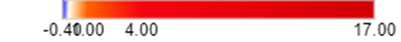

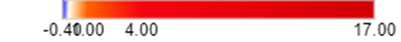

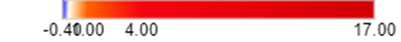

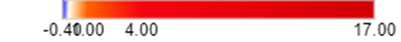


**0**

**16**

**4**

**1**

**-0.4**

**Column Z score**

**Figure. S5**

**Figure S5. Verification of Knockdown Efficacy.** The mRNA expression levels of candidate transcription factors (TFs) in established knockdown cell lines were quantified. Relative mRNA expression levels were determined using the 2^(-ΔΔCt) method, with GAPDH serving as the internal reference gene. Statistical significance is indicated as follows: *, P < 0.05; **, P < 0.01; ***, P < 0.001; ****, P < 0.0001.

**
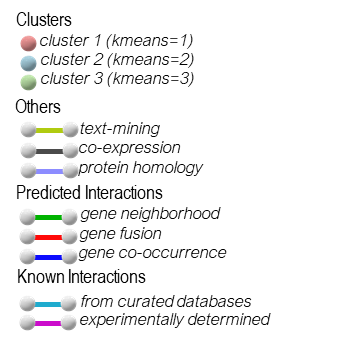
**
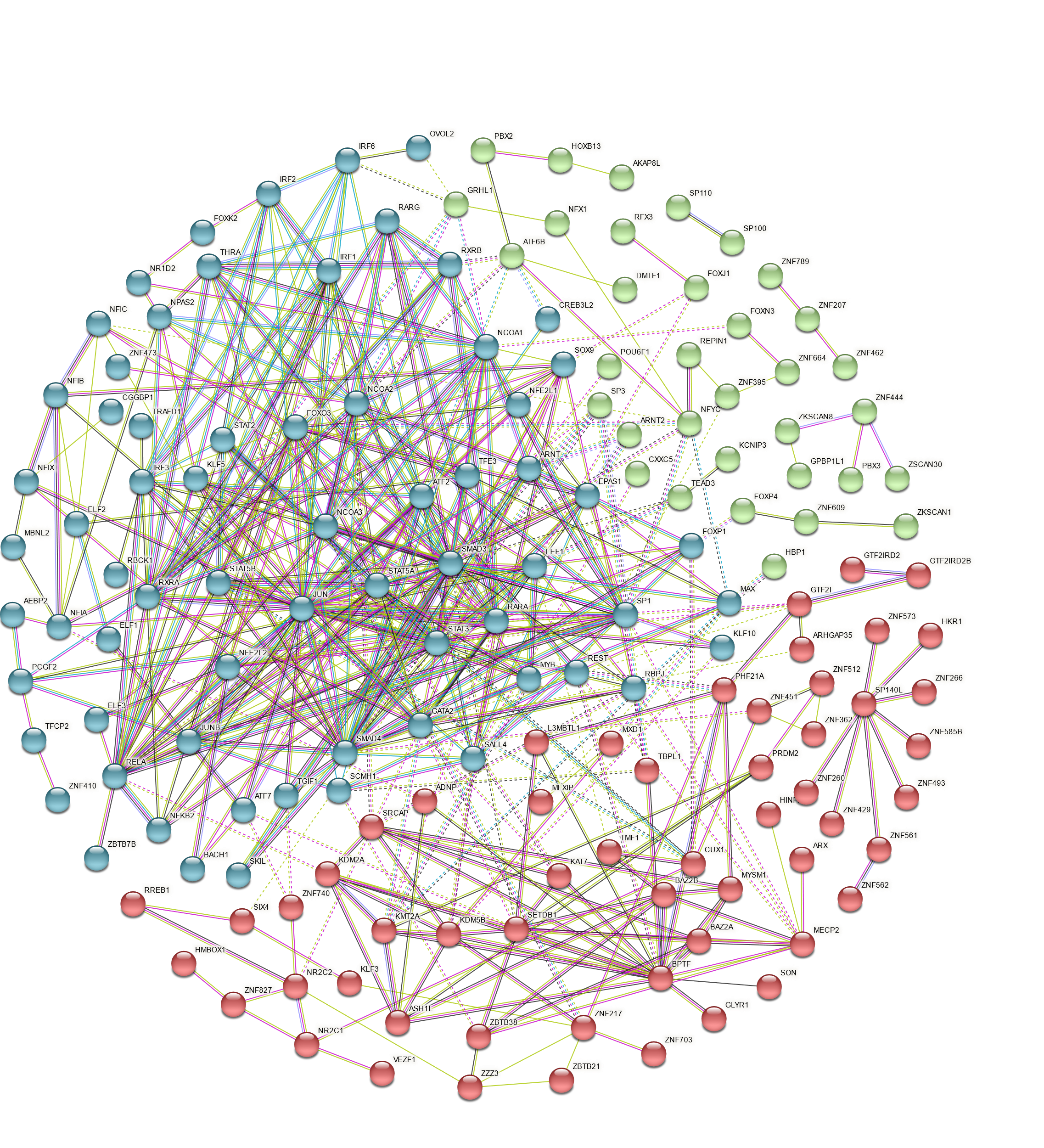
**Figure. S6**

**Figure S6. The STRING protein-protein interaction (PPI) network among dormant-TFs.** The network diagram illustrates the protein-protein interactions (PPIs) among dormant TFs, identified through the STRING database. Each node in the network represents a TF, with edges between nodes indicating interactions between TFs as detailed in the legend. Clusters of densely interconnected nodes signify distinct protein complexes or functional modules.
